# BsmR degrades c-di-GMP to modulate biofilm formation of nosocomial pathogen *Stenotrophomonas maltophilia*

**DOI:** 10.1038/s41598-017-04763-w

**Published:** 2017-07-05

**Authors:** Wei Liu, Xiu-Qi Tian, Jin-Wei Wei, Li-Li Ding, Wei Qian, Zhong Liu, Fang-Fang Wang

**Affiliations:** 1 School of Pharmacy, Shanghai Jiao Tong University, Shanghai, 200240 China; 2 State Key Laboratory of Plant Genomics, Institute of Microbiology, Chinese Academy of Sciences, Beijing, 100101 China; 3 School of Life Sciences, University of Chinese Academy of Sciences, Beijing, 100049 China

**Keywords:** Gene regulation, Bacterial genetics

## Abstract

c-di-GMP is a cellular second messenger that regulates diverse bacterial processes, including swimming, biofilm formation and virulence. However, in *Stenotrophomonas maltophilia*, a nosocomial pathogen that frequently infects immunodeficient or immunoincompetent patients, the regulatory function of c-di-GMP remains unclear. Here we show that BsmR is a negative regulator of biofilm development that degrades c-di-GMP through its EAL domain. Increasing BsmR expression resulted in significant increase in bacterial swimming and decrease in cell aggregation. BsmR regulates the expression of at least 349 genes. Among them, 34 involved in flagellar assembly and a flagellar-assembly-related transcription factor (*fsnR*) are positively regulated. Although BsmR is a response regulator of the two-component signaling system, its role in biofilm formation depends on the expression level of its respective gene (*bsmR*), not on the protein’s phosphorylation level. A transcription factor, BsmT, whose coding gene is located in the same tetra-cistronic operon as *bsmR*, was shown to directly bind to the promoter region of the operon and, through a positive regulatory loop, modulate *bsmR* transcription. Thus, our results revealed that the c-di-GMP signaling pathway controls biofilm formation and swimming in *S. maltophilia*, suggesting c-di-GMP signaling as a target in the development of novel antibacterial agents to resist this pathogen.

## Introduction

*Stenotrophomonas maltophilia* is a gram-negative bacterium belonging to the family Xanthomonadaceae. As an obligate aerobe it thrives in diverse ecological niches, including foods, soil, water systems, and plant roots^1, 2^. Moreover, it can persist for prolonged periods even in nutrient-poor aqueous environments^3–6^. In recent years, *S. maltophilia* has been identified not only as a globally emerging pathogen but also as an important opportunistic pathogen in hospital-acquired infections, accounting for ~65% of nosocomial infections^7^. A key element of the resilience and difficult eradication of *S. maltophilia* is the formidable capability of this bacterium to form biofilm, defined as a bacterial aggregate colonizing solid–liquid interfaces^8, 9^. Compared with planktonic cells, the bacteria embedded in a biofilm are 10–1,000 times more resistant to antimicrobial agents^10–12^. Biofilms of *S. maltophilia* have been found in various clinical settings and medical implants, mostly in immunocompromised or immunosuppressed hospitalized patients, and especially those in the intensive care unit (ICU). Mortality rates from *S. maltophilia* infections range from 23% to 77%^9, 13–20^. Although the World Health Organization recommends the treatment of these infections with trimethoprim-sulfamethoxazole, the susceptibility of *S. maltophilia* has rapidly decreased over the space of a decade, from >98% to 30–40%^8, 21, 22^. An effective antimicrobial strategy to combat *S. maltophilia* infections would be to interfere with its ability to form biofilms. However, the success of this approach depends on an understanding of the regulatory mechanisms in *S. maltophilia* biofilm development.

The environmental factors affecting *S. maltophilia* biofilm formation have been investigated, but the molecular basis of their regulatory mechanisms remains incompletely understood^23–26^ and thus far only a few related *S. maltophilia* genes have been experimentally studied. For example, several structural genes associated with the cell envelope, including those encoding the proteins involved in lipopolysaccharide/exopolysaccharide-coupled biosynthesis (*rmlA*, *rmlC*, and *xanB*) and the pump-encoding genes *macABCsm* and *smeYZ* have been identified as necessary for biofilm formation^27–29^. In addition, the genes encoding three transcription regulators (*fleQ*, *fsnR*, and *bfmA*) also control biofilm development. FleQ binds to the putative ATPase FleN to form a complex that directs flagellar gene expression^30^. FsnR, designated as a response regulator with transcription-regulating activity, binds directly to the promoter regions of gene clusters involved in flagellar assembly to activate their transcriptional initiation^31, 32^.

Besides the aforementioned regulatory factors, recent studies have identified bis-3′, 5′-cyclic diguanosine monophosphate (c-di-GMP) as an important cellular second messenger broadly distributed among bacteria and critical to the control of bacterial physiology, especially biofilm development and motility^33^. c-di-GMP activates downstream cascades by binding to specific protein effectors or riboswitches embedded in the leader regions of mRNAs. The turnover of intracellular c-di-GMP concentrations is modulated by two classes of enzymes, diguanylate cyclases (DGCs) and phosphodiesterases (PDEs). DGCs are GGDEF-domain-containing proteins that catalyze c-di-GMP biosynthesis from two molecules of GTP. PDEs are EAL- or HD-GYP-domain-containing proteins catalyzing the hydrolysis of c-di-GMP into pGpG or GMP. The processes associated with c-di-GMP turnover have been extensively documented in various animal pathogens, but none of the 27 GGDEF, EAL, or HD-GYP proteins (Supplementary Fig. S1) of *S. maltophilia* have been experimentally investigated. Their regulatory functions during biofilm development are therefore completely unknown.

**Figure 1:**
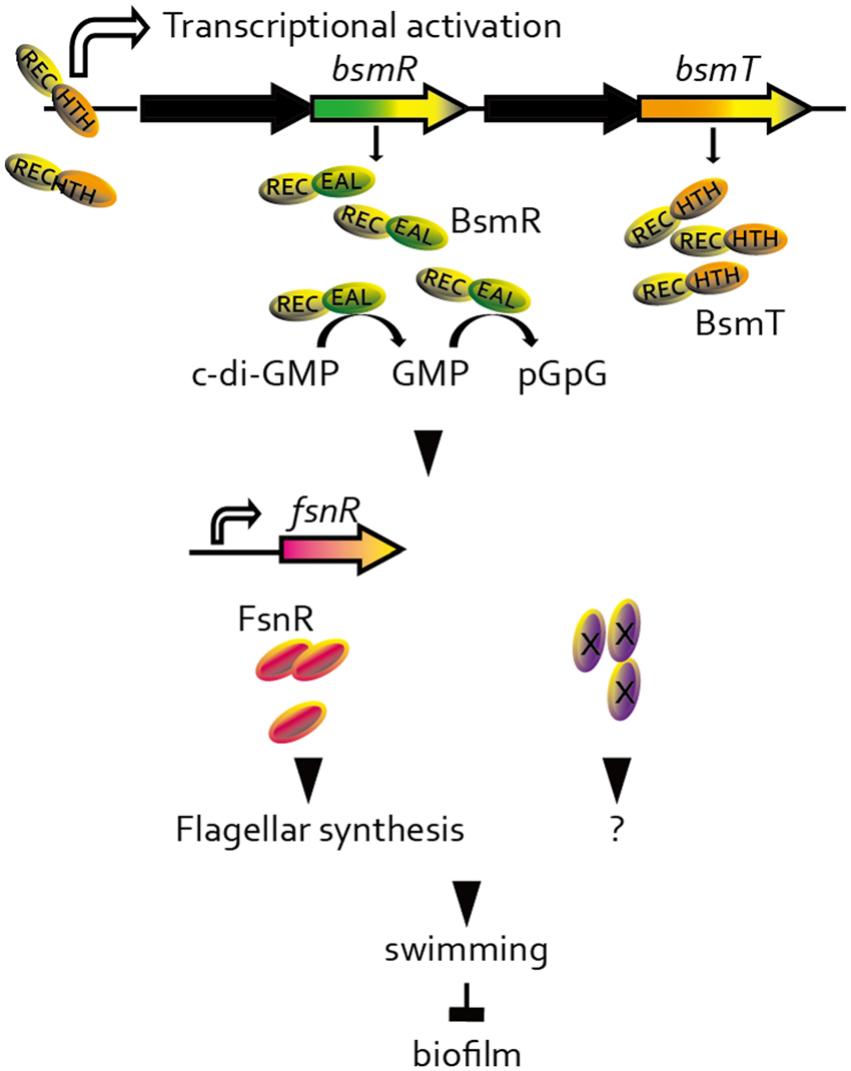
Working model. BsmR degrades c-di-GMP to decrease its cellular level, which activates the expression of FsnR, a transcriptional regulator controlling flagellar synthesis, to promote bacterial swimming motility and diminish the biofilm formation. Although as a response regulator, the PDE activity and the regulatory role of BsmR on bacterial swimming motility and biofilm formation do not depend on the phosphorylation level of BsmR but on its expression level, which is controlled by BsmT, another response regulator encoded by the *bsmR* operon. BsmT, working as a transcriptional regulator, directly binds to the promoter region of the *bsmR* operon and positively regulates the expression of all four genes in the *bsmR* operon, including *bsmR*. REC: conserved receiver domain; HTH: helix-turn-helix DNA binding domain; EAL: EAL domain; hallow arrows: transcription activation; ×: unknown regulatory elements;?: unknown regulatory role of ×.

In this study, we demonstrated the role of BsmR, an EAL-domain-containing response regulator (RR) in *S. maltophilia* CGMCC 1.1788, in controlling biofilm formation and swimming motility. Gradient increase in the expression of *bsmR* resulted in gradient increase in swimming motility and decrease in biofilm formation. BsmR degrades c-di-GMP to activate the expression of the transcriptional regulator of FsnR, which positively controls the transcription of two flagellar synthesis gene clusters to promote bacterial swimming motility. Unlike other RRs, the PDE activity of BsmR is independent of the protein’s phosphorylation level but does depend on the transcriptional level of *bsmR*. The latter is controlled by BsmT, encoded by a gene located in the same operon as *bsmR*. By autoregulation, BsmT directly binds to the promoter region of its own operon and activates *bsmR* expression (Fig. 1). In the present study, we dissected a c-di-GMP signaling cascade to show that this second messenger also regulates *S. maltophilia* biofilm formation. Our results will facilitate the development of new approaches to interfere with *S. maltophilia* biofilm formation, by targeting the c-di-GMP pathway.

## Results

### BsmR controls bacterial swimming motility and biofilm formation independent of its phosphorylation level

**Figure 2:**
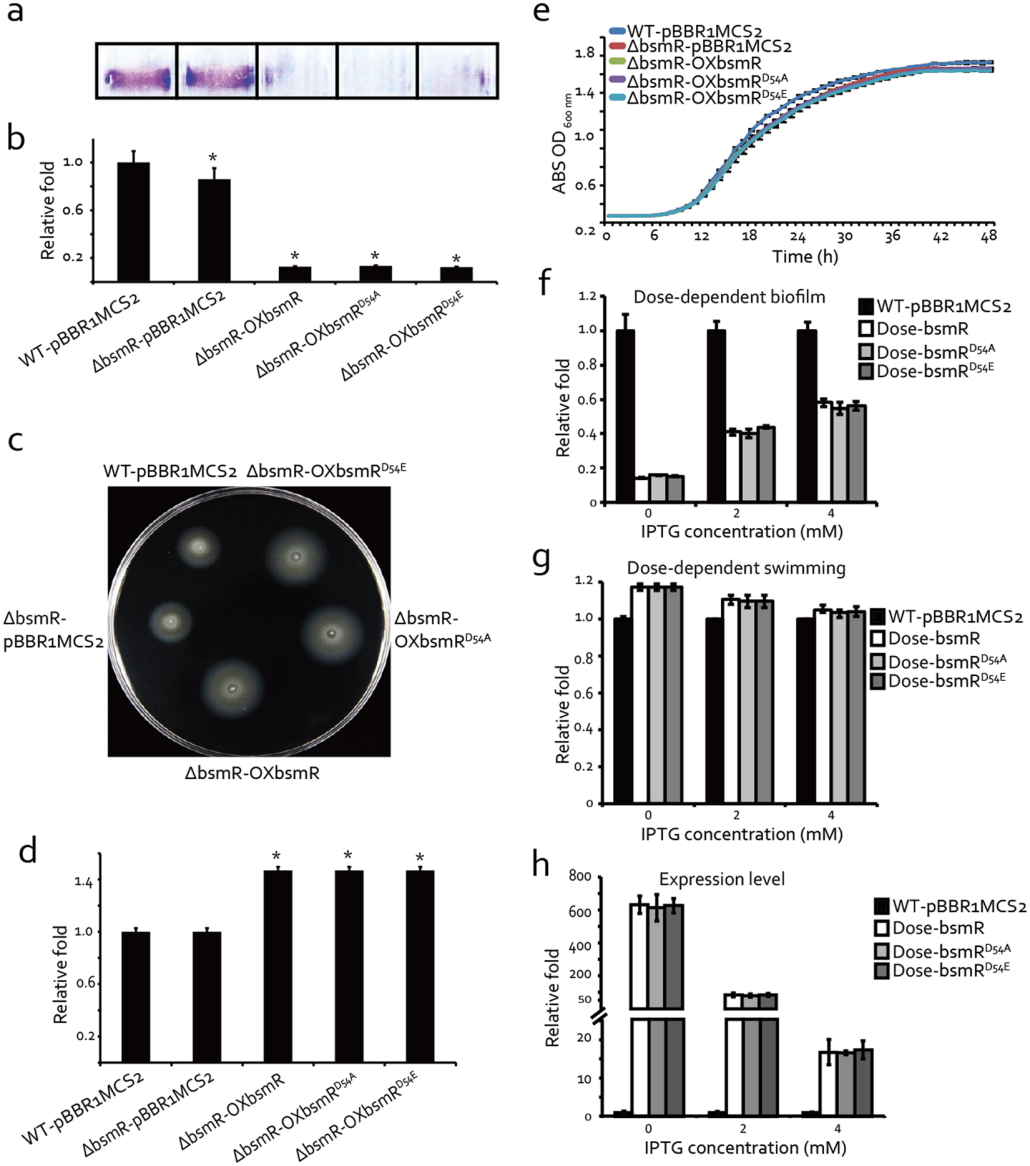
*bsmR* negatively regulates bacterial swimming motility and diminishes biofilm formation. (**a**) Biofilms formed by different bacterial strains and evaluated by crystal violet staining. (**b**) Relative quantification of the biofilm in (**a**) at 590 nm using a Tecan Infinite 200 Pro scanner. The experiment was repeated 4 times. (**c**) Swimming motility of different bacterial strains in rich NYG medium containing 0.15% agar. (**d**) Relative quantification of the diameters of the bacterial swimming zones. The data are the average of three separate measurements. (**e**) Growth curves of bacterial strains grown in rich NYG medium at 28 °C. ABS: absorbance. (**f**) Dose-dependent effect on biofilm formation. Relative quantification of the biofilm was measured when different concentrations of IPTG was used to suppress the expression of *bsmR/bsmR* ^*D54A*^/*bsmR* ^*D54E*^. (**g**) Dose-dependent effect on the swimming motility. Relative quantification of the diameters of the swimming zones was measured when different concentrations of IPTG was used to suppress the expression of *bsmR/bsmR* ^*D54A*^ */bsmR* ^*D54E*^. (**h**) The expression level of *bsmR/bsmR* ^*D54A*^ */bsmR* ^*D54E*^ along with the addition of IPTG. Relative quantification of *bsmR/bsmR* ^*D54A*^ */bsmR* ^*D54E*^ expression level using qRT-PCR assays. WT-pBBR1MCS2: wild-type strain containing the blank pBBR1MCS2 vector; ΔbsmR-pBBR1MCS2: *bsmR* in-frame deletion mutant containing the blank pBBR1MCS2 vector; ΔbsmR-OXbsmR: *bsmR* in-frame deletion mutant containing the recombinant pBBR1MCS2-bsmR vector; ΔbsmR-OXbsmR^D54A^: *bsmR* in-frame deletion mutant containing the recombinant pBBR1MCS2-bsmR^D54A^ vector; ΔbsmR-OXbsmR^D54E^: *bsmR* in-frame deletion mutant containing the recombinant pBBR1MCS2-bsmR^D54E^ vector; Dose-bsmR: *bsmR* in-frame deletion mutant containing the recombinant MpBBR1MCS2-bsmR; Dose-bsmR^D54E^: *bsmR* in-frame deletion mutant containing the recombinant MpBBR1MCS2-bsmR^D54E^; Dose-bsmR^D54A^: *bsmR* in-frame deletion mutant containing the recombinant MpBBR1MCS2-bsmR^D54A^. All data are representatives of at least triplicate repeatable experiments. All values are the means ± standard deviations. *p < 0.05, as determined by ANOVA.

Bioinformatics analysis revealed that the *S. maltophilia* ATCC 13637 genome encodes 27 proteins involved in c-di-GMP metabolism, including 18 GGDEF-domain-containing proteins, 3 EAL domain-containing proteins, and 6 proteins containing both the GGDEF and EAL domains (Supplementary Fig. S1). Among them, DP16_RS18245 contains an N-terminal REC domain and a C-terminal EAL domain, marking it as a putative RR with diguanylate PDE activity. A mutational analysis demonstrated that *DP16_RS18245* is involved in bacterial swimming motility and the regulation of biofilm formation. As shown in Fig. 2a–d, an in-frame deletion of this gene (strain ΔbsmR) did not cause recognizable phenotypic changes in biofilm formation or swimming motility. However, the overexpression of this mutant gene in *S. maltophilia*, achieved by transforming strain ΔbsmR with a recombinant broad-host-range plasmid (pBBR1MCS2::*DP16_RS18245*), yielding strain ΔbsmR-OXbsmR whose expression level of *bsmR* was 4255 fold of that of the WT strain (Fig. S3), resulted in a significant increase in the bacterial swimming zone, to 152.2% of the level of the WT strain. Overexpression also led to a decrease in biofilm development, to 4.0% of the WT strain level. In addition, the regulatory role of *bsmR* on biofilm formation and swimming motility was specific since overexpression of any other EAL-domain containing proteins encoded by *S. maltophillia ATCC 13637* did not remarkably decreased the biofilm formation or increased the swimming motility, although some slight but significant decrease in the biofilm formation and increase in the swimming motility were identified (Fig. S2). The growth of both the mutant (ΔbsmR) and the overexpressing (ΔbsmR-OXbsmR) strains was similar to that of the WT strain, indicating that the phenotypic alterations were not caused by differences in growth rates (Fig. 2e). Together, these results suggested that *DP16_RS18245* is a negative regulator of biofilm formation and a positive regulator of bacterial swimming motility. Therefore, *DP16_RS18245* was named *bsmR* (biofilm and swimming motility regulator). Considering the possibility that overexpression systems might cause artificial phenotypes, a dose-dependent assay was used to verify the role of BsmR. Strains used in this assay were constructed by transforming the recombinant plasmid MpBBR1MCS2::*bsmR* which was achieved by replacing the *lacZ* promoter of pBBR1MCS2::*bsmR* with *araC* gene and the arabinose *P*_*BAD*_ promoter, into ΔbsmR strain. As shown in Fig. 2f–h, the gradient increase in biofilm quantity and the gradient decrease in swimming zones were identified when the gradient concentration of IPTG was used to suppress the activity of the arabinose *P*_*BAD*_ promoter. Parallelly, the expression level of *bsmR* was shown to be a gradient decrease accompanying the increase in IPTG concentration. The results of the dose-dependent assay verified the roles for *bsmR* as a negative regulator of biofilm formation and a positive regulator of swimming motility.

In proteins of the two-component signal transduction system (TCS), the REC domain of a RR contains an invariant Asp residue as a phosphorylation site, and the level of RR phosphorylation is usually essential for TCS regulation^34^. To genetically determine the role of phosphorylation in BsmR regulation, two *S. maltophilia* recombinant strains were constructed using the ΔbsmR background: Strain ΔbsmR-OXbsmR^D54A^ was constructed by transforming ΔbsmR with the pBBR1MCS2::bsmR^D54A^ plasmid, in which nucleotides encoding the phosphorylation site Asp^54^ were substituted by those encoding an Ala residue. In this strain, the dephosphorylation of BsmR was constitutive. In strain ΔbsmR-OXbsmR^D54E^, pBBR1MCS2::bsmR^D54E^, in which the phosphorylation site was altered to encode a Glu, resulted in the constitutive phosphorylation of BsmR. Genetic analyses showed that the properties of the two recombinant strains with respect to biofilm development and bacterial swimming were similar to those of the *bsmR*-overexpressing strain (ΔbsmR-OXbsmR) (Fig. 2a–d). In addition, no significant difference was identified in the dose-dependent assays between the two recombinant strains and the *bsmR*-overexpressing strain (Fig. 2f–h). Collectively, these results indicated that BsmR controls both bacterial swimming motility and biofilm formation in a phosphorylation-independent manner.

### *bsmR* is located in a tetra-cistronic operon

**Figure 3:**
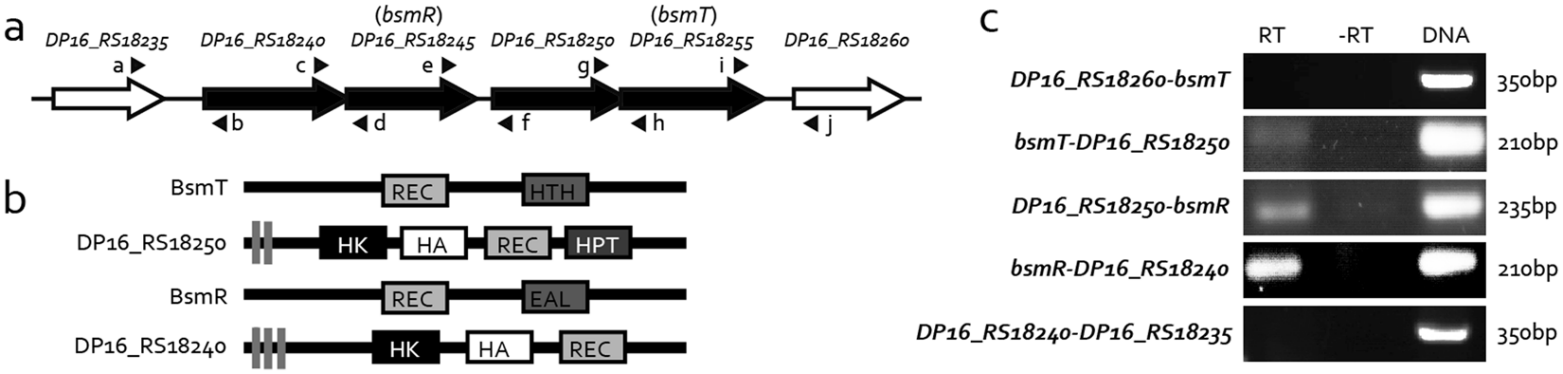
RT-PCR analyses demonstrate *DP16_RS18240*-*bsmR*-*DP16_RS18250*-*bsmT* as a tetra-cistronic operon. (**a**) Genomic localization of the *bsmR* gene. Arrows indicate the genes and their transcriptional directions. The black arrows indicate genes predicted to be in an operon. Black triangles around the arrows indicate the positions of the primers used in the RT-PCR assay. Gene names are listed above. (**b**) Putative secondary protein structures of BsmT, DP16_RS18250, DP16_RS18240, and BsmR. Protein structures were predicted by searching the Pfam database. (**c**) *bsmR* operon structure analysis by RT-PCR. RT: Amplification was carried out using cDNA transcribed from total RNAs as the template. −RT: cDNA synthesis in the absence of reverse transcriptase (negative control); DNA: PCR amplification with total DNA of *S. maltophilia* as the template (positive control). The data are representatives of triplicate repeatable experiments.

In bacteria, genes located in an operon are usually functionally associated^35–37^. According to the operon structure algorithms DOOR^38^ and MicrobesOnline Operon Predictions^39, 40^, *bsmR* was predicted to be in a genomic neighborhood that included a putative operon containing three other genes: *DP16_RS18255*, *DP16_RS18250*, and *DP16_RS18240* (Fig. 3a). To verify the prediction, RT-PCR analyses were employed to detect transcripts generated from the intergenic regions between these genes. In repeated experiments, stable RT-PCR products representing positive amplification of the intergenic transcripts between *DP16_RS18255-DP16_RS18250, DP16_RS18250-bsmR*, and *bsmR-DP16_RS18240* were identified (Fig. 3c), supporting the inclusion of these four genes within an operon (*DP16_RS18255-DP16_RS18250-bsmR-DP16_RS18240*).

Among the four proteins encoded by this operon, DP16_RS18255 (named BsmT, biofilm and swimming motility related transcriptional regulator) is a RR with a typical REC domain and a helix-turn-helix domain. It is a putative transcription factor. Both DP16_RS18250 and DP16_RS18240 are histidine kinases (HKs) with conserved transmitter domains (Fig. 3b). In bacteria, a HK and its cognate RR constitute a typical TCS, the dominant bacterial signaling system. Since paired HK-RRs are usually encoded by neighboring genes in the genome^34, 41^, we proposed that the four operon-encoded proteins constitute two TCSs. To verify this hypothesis, four C-terminal His_6_-tagged recombinant proteins, BsmT-His_6_, DP16_RS18250-His_6_, BsmR-His_6_, and DP16_RS18240-His_6_, were expressed and then purified by Ni-nitrilotriacetic acid (Ni-NTA) affinity chromatography for use in an *in vitro* phosphorylation assay. However, under the assay conditions, two HKs (DS16_RS18250 and DP16_RS18240) failed to yield a phosphorylation band or phosphorylate BsmR or BsmT (Supplementary Fig. S5). These results suggested that the half-life of their phosphorylated state was too short to be detected or that unknown factors are necessary to activate their phosphorylation or stabilize their phosphorylation states, which should be investigated in the future study.

### *bsmR* modulates cellular c-di-GMP levels to control bacterial swimming motility and biofilm formation

**Figure 4:**
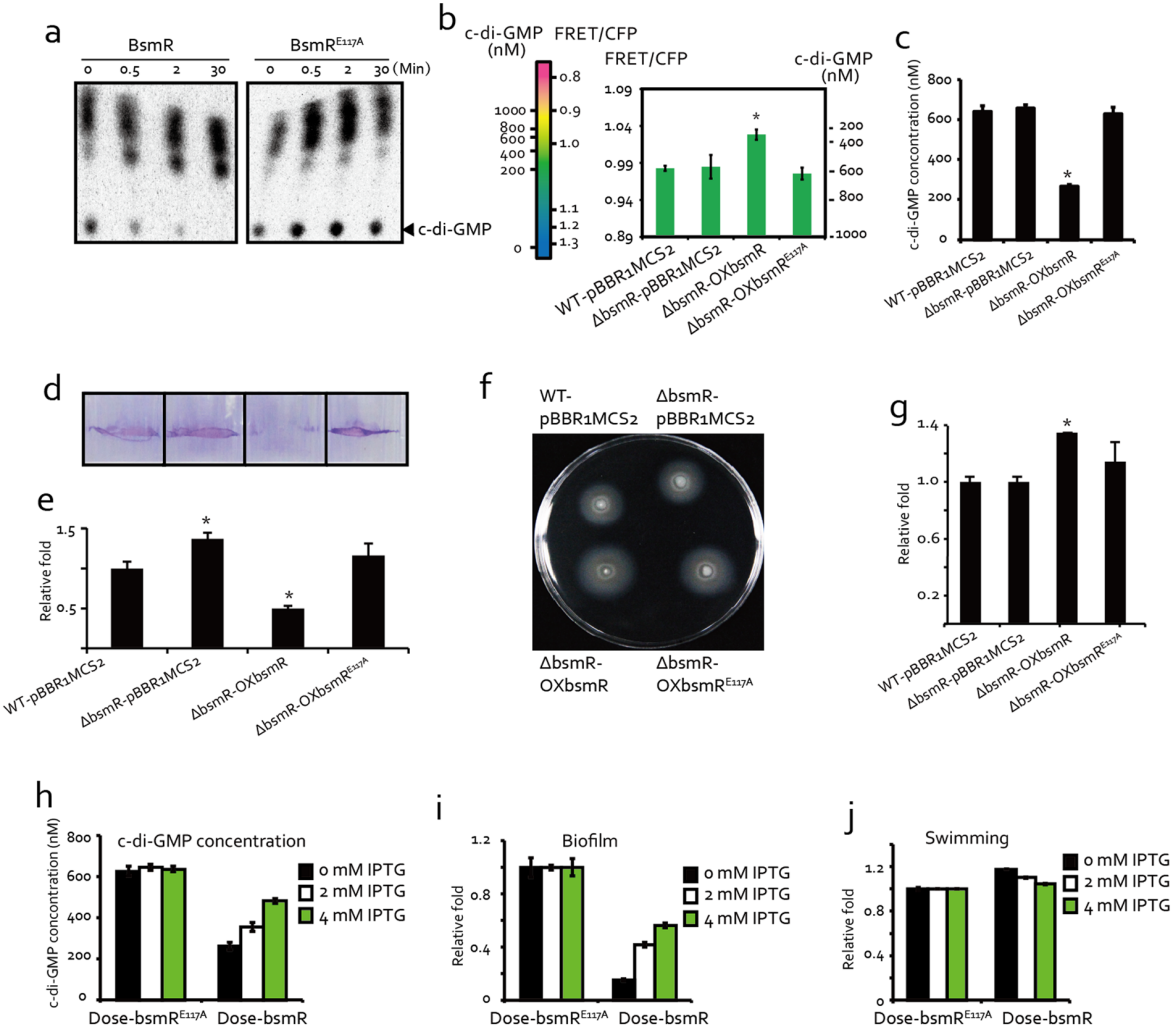
BsmR degrades c-di-GMP to regulate bacterial swimming motility and biofilm formation. (**a**) BsmR protein degrades c-di-GMP *in vitro*. Purified BsmR protein was incubated at 28 °C in reaction buffer together with the synthesized ^32^P-labeled c-di-GMP. At the indicated time points, aliquots of equal volume were retrieved and separated by thin layer chromatography. (**b**) Overexpressing *bsmR* significantly decreased the cellular c-di-GMP level. The FRET-based c-di-GMP biosensor was used to measure the amounts of intracellular c-di-GMP. The table of FRET values and c-di-GMP concentrations was generated by incubating the purified c-di-GMP biosensor with increasing concentrations of c-di-GMP. (**c**) Quantification of cellular c-di-GMP level by LC-MS/MS. (**d**) Biofilm of the bacterial strains stained with crystal violet. (**e**) Quantification of the relative bacterial levels in the biofilm shown in (**d**). (**f**) Swimming motility of the indicated strains grown on rich NYG medium plates containing 0.15% agar. (**g**) Relative quantification of bacterial swimming zone diameters. (**h**) Quantification of the cellular c-di-GMP level of strains induced by different concentrations of IPTG. (**i**) Relative quantification of the biofilm formed by strains induced by different concentrations of IPTG. (**j**) Relative quantification of the diameters of the swimming zones formed by strains induced by different concentrations of IPTG. The data are representatives of three independent experiments. Each value is the average of three replicates. Error bars represent the standard deviation (n ≥ 3). *p < 0.05, as determined by AVONA. The data are representative of at least triplicate repeatable experiments.

The N-terminal EAL domain of BsmR has putative PDE activity and was therefore expected to degrade the bacterial second messenger c-di-GMP. This finding was confirmed by expressing and then purifying recombinant BsmR-His_6_ for use in an *in vitro* c-di-GMP degradation assay. BsmR-His_6_ proteins were incubated with synthesized ^32^P-labeled c-di-GMP and the reactions were stopped at specific time points by the addition of an equal volume of 0.5 mM EDTA. The c-di-GMP levels in the reactions were then analyzed by thin layer chromatography. As shown in Fig. 4a, BsmR-His_6_ gradually degraded c-di-GMP such that after 30 min the c-di-GMP in the reaction was completely hydrolyzed to GMP. However, substitution of the active residue (Glu^117^) in the EAL domain by an Ala (recombinant protein BsmR^E117A^-His_6_) completely eliminated the PDE activity of BsmR. By adopting a FRET-based biosensor approach we were able to determine the concentration of c-di-GMP in bacterial cells. The FRET-based biosensor YFP-YcgR-CFP, in which the c-di-GMP receptor YcgR was inserted between CFP and YFP (cyan and yellow fluorescent proteins)^42, 43^, was used to transform *Sma* strains via the broad-host-range vector pBBR1MCS1 (recombinant plasmid pBBR1MCS1::yfp-ycgR-cfp). Binding of the biosensor to cellular c-di-GMP induced a conformational change that decreased FRET efficiency. The cellular c-di-GMP concentration was then determined by comparing the FRET efficiency of the cellular level with that of the *in vitro* YFP-YcgR-CFP protein in reactions with various concentrations of c-di-GMP. As shown in Fig. 4b, the overexpression of BsmR significantly decreased the cellular c-di-GMP level, to 50% of that of the WT strain, whereas there was no recognizable impact by overexpressing the inactive form of BsmR (BsmR^E117A^), which was consistent with the results of the cellular c-di-GMP level quantification by LC-MS/MS (Fig. 4c). Additionally, the gradual increase in cellular c-di-GMP level was identified when series concentrations of IPTG ranging from 0 to 4 mM were used to suppress the expression of *bsmR* (Fig. 4h). These results provide *in vitro* and *in vivo* evidence that BsmR is a PDE able to degrade c-di-GMP via its EAL domain.

To determine whether the PDE of BsmR is an essential regulator of bacterial swimming motility and biofilm formation, a strain overexpressing the inactive form of BsmR (ΔbsmR-OXbsmR^E117A^) was constructed using the genetic background of the *bsmR* mutant. As shown in Fig. 4d–g, compared with the in-frame deletion mutant strain of *bsmR*, biofilm formation and bacterial swimming of ΔbsmR-OXbsmR^E117A^ strain cells were unaffected; in fact, the levels of both were similar to those of the WT strain. By contrast, the overexpression of *bsmR* resulted in significant alterations in both phenotypes. Additionally, the dose-dependent effects of cellular c-di-GMP level controlled by BsmR were also detected. As shown in Fig. 4h–j, the cellular c-di-GMP level was gradually increased along with the addition of increasing concentrations of IPTG to suppress the BsmR expression level. Parallelly, the gradient increase in biofilm quantities and decrease in biofilm swimming zones were identified. On the contrary, no dose-dependent effects of BsmR^E117A^ were identified. These results suggested that the degradation of cellular c-di-GMP by BsmR is critical to the regulation of swimming and cell aggregation of *S. maltophilia*.

### Dissection of the *bsmR* regulon by high-throughput RNA sequencing (RNA-seq)

**Figure 5:**
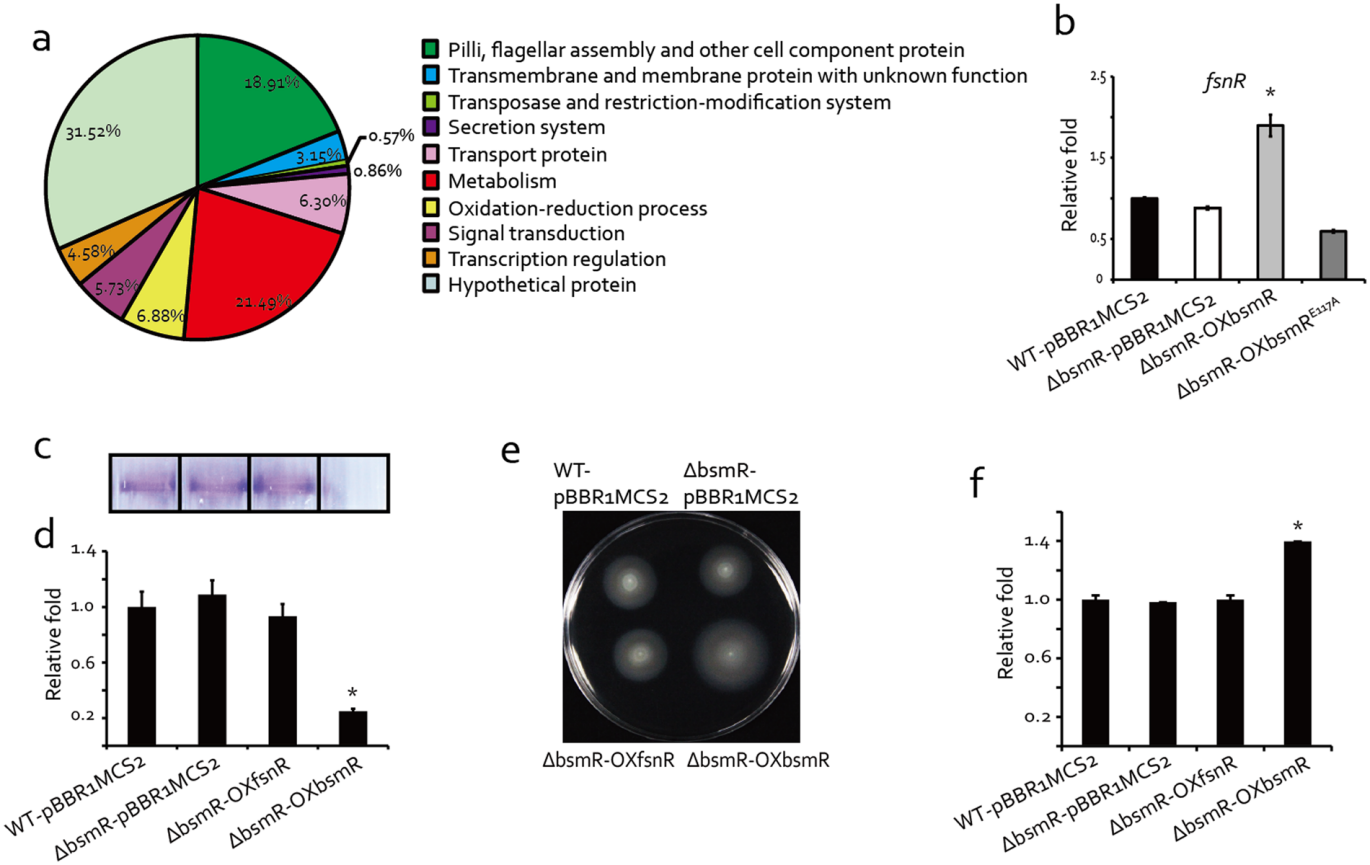
Dissection of the BsmR regulon by RNA-seq. (**a**) *bsmR* regulon analysis by high-throughput RNA sequencing. The putative target genes were functionally categorized into 10 groups (Supplementary Table S3). (**b**) qRT-PCR assay shows the positive regulation of *fsnR* by *bsmR*. cDNA was reverse-transcribed from the total RNAs of the indicated bacterial strains. The tmRNA level served as the internal control. (**c**) A biofilm formed by the bacterial strains and stained with crystal violet. (**d**) Relative quantification of the biofilm levels of the bacterial strains. (**e**) Swimming motility of the indicated bacterial strains on rich NYG medium plates containing 0.15% agar. (**f**) Relative quantification of bacterial swimming zone diameters. Each value is the average of three replicates. Error bars represent standard deviation (n ≥ 3). *p < 0.05, as determined by ANOVA. The data are representatives of at least triplicate repeatable experiments.

To understand how BsmR regulates bacterial swimming and biofilm formation, RNA-seq analysis was used to dissect the *bsmR* regulon. Genome-wide transcription levels in WT-pBBR1MCS2 and ΔbsmR-OXbsmR strains were compared as follows. Total RNAs were extracted from bacterial cells grown in rich NYG medium to an optical density at 600 nm (OD_600_) of 0.4 and used to generate cDNA by reverse transcription with random hexamer primers. The newly generated cDNAs were used for adaptor ligation and subsequent PCR amplification, followed by high-throughput Illumina sequencing. A comparison of the gene expression levels of the WT-pBBR1MCS2 and ΔbsmR-OXbsmR strains revealed that the overexpression of *bsmR* caused the up-regulation of 282 genes and the down-regulation of 67 genes (threshold > 2 fold, Fig. 5a and Supplementary Table S3). To verify the results, qRT-PCR assays were used to determine the expression level of *bsmR* and 9 other randomly selected genes among the putative target genes of *bsmR*. As shown in Fig. S3, in the overexpression strain (ΔbsmR-OXbsmR), the expression level of *bsmR* was 4255 fold of that in the WT-pBBR1MCS2 strain. Besides, 8 genes, including DP16_RS11225, DP16_RS11175, DP16_RS11180, DP16_RS11185, DP16_RS11190, DP16_RS11195, DP16_RS11260 and DP16_RS11360 were significantly up-regulated and 1 gene, DP16_RS09460, was significantly down-regulated in the overexpression strain (ΔbsmR-OXbsmR), which was consistent with the results of RNA-seq.

The 349 genes identified by RNA-seq analysis were functionally classified into 10 groups: cell component proteins, transmembrane and membrane proteins, transposase and restriction-modification, secretion, transport proteins, metabolism, oxidation-reduction, signal transduction, transcriptional regulation, and hypothetical proteins (Fig. 5a and Supplementary Table S3). Except the largest group, hypothetical proteins, containing 110 genes with a proportion of 31.52% of the genes regulated by BsmR under the assay conditions, there are two larger groups, metabolism proteins and cell component proteins, suggesting that BsmR plays an important role in modulating the cellular biophysical and biochemical reactions as well as cell component synthesis and assembly, which is the precondition to alter bacterial behavior. It was noteworthy that 34 genes associated with flagellar assembly (ratio 34/39, namely 87.2% of the genes involved in flagellar assembly in *S. maltophilia* ATCC 13637) were up-regulated in the *bsmR*-overexpressing strain, which suggested that *bsmR* positively controls bacterial flagellar assembly to modulate swimming motility. Additionally, in the signal transduction group, 12 chemotaxis-related genes, including the up-regulated chemotaxis gene cluster *cheA-cheW-cheB-cheY-cheZ*, were identified. Previous studies demonstrated that the chemotaxis signaling pathway controls the flagellar motor as well as bacterial motility^44, 45^. For example, in *Escherichia coli*, phosphorylated CheY binds to FliM, a switch protein located at the base of the flagellar body, to shift the direction of flagellar rotation from counterclockwise to clockwise^46, 47^. Taken together, these findings are consistent with the role for BsmR as a regulator of bacterial motility, by controlling both flagellar assembly and flagellar motor movements. Furthermore, a group of 24 genes involved in oxidation-reduction process were shown to be regulated by BsmR (Fig. 5a and Supplementary Table S3), which suggested another role for BsmR as a modulator of the cellular redox state. Besides the functional genes, some regulatory genes were also identified, including 4 sigma factors and 11 transcriptional regulators (Supplementary Table S3). Among them, there is a response regulator coding gene, *DP16_RS11195*, previously reported to encode the transcriptional regulator FsnR^32^. This positive regulator of bacterial swimming mobility directly binds to the promoter sequences of 10 flagella-associated genes to activate their transcription: *DP16_RS19075*, *DP16_RS11295* (*flhB*), *DP16_RS11250*, *DP16_RS11215* (*fliE*), *DP16_RS11200*, *DP16_RS11175* (*flgD*), *DP16_RS11160* (*flgC*), *DP16_RS11100*, *DP16_RS11095* (*flgA*), and *DP16_RS11070*. We therefore hypothesized that BsmR activates *fsnR* transcription to modulate the activity of these genes. As shown in Fig. 5b, overexpressing *bsmR* significantly increased the *fsnR* mRNA level to 189.6% of that of the WT strain (Fig. 5b), indicating that *fsnR* is positively regulated by BsmR. However, epistasis analyses showed that the overexpression of *fsnR* against the *bsm*R in-frame deletion background had no influence on bacterial swimming motility or biofilm formation (Fig. 5c–f). This was probably because, besides *fsnR*, which modulates only a small portion of *bsmR*-targeted flagella-related genes (ratio 10/34), other transcriptional regulators or regulatory events might be activated simultaneously to cooperatively modulate bacterial motility and biofilm formation. For example, FliA, one of the sigma factors positively regulated by BsmR, was reported to modulate the swimming and biofilm formation by controlling the flagellum number in *Rhodobacter sphaeroides* ^48^. And DP16_RS09435, a transcriptional regulator negatively regulated by BsmR, is homologous to the CRP/FNR family protein which is a c-di-GMP effector reported to regulate biofilm formation in *Burkholderia cenocepacia* ^49^.

### *bsmT* is involved in the regulation of bacterial swimming motility and biofilm formation

**Figure 6:**
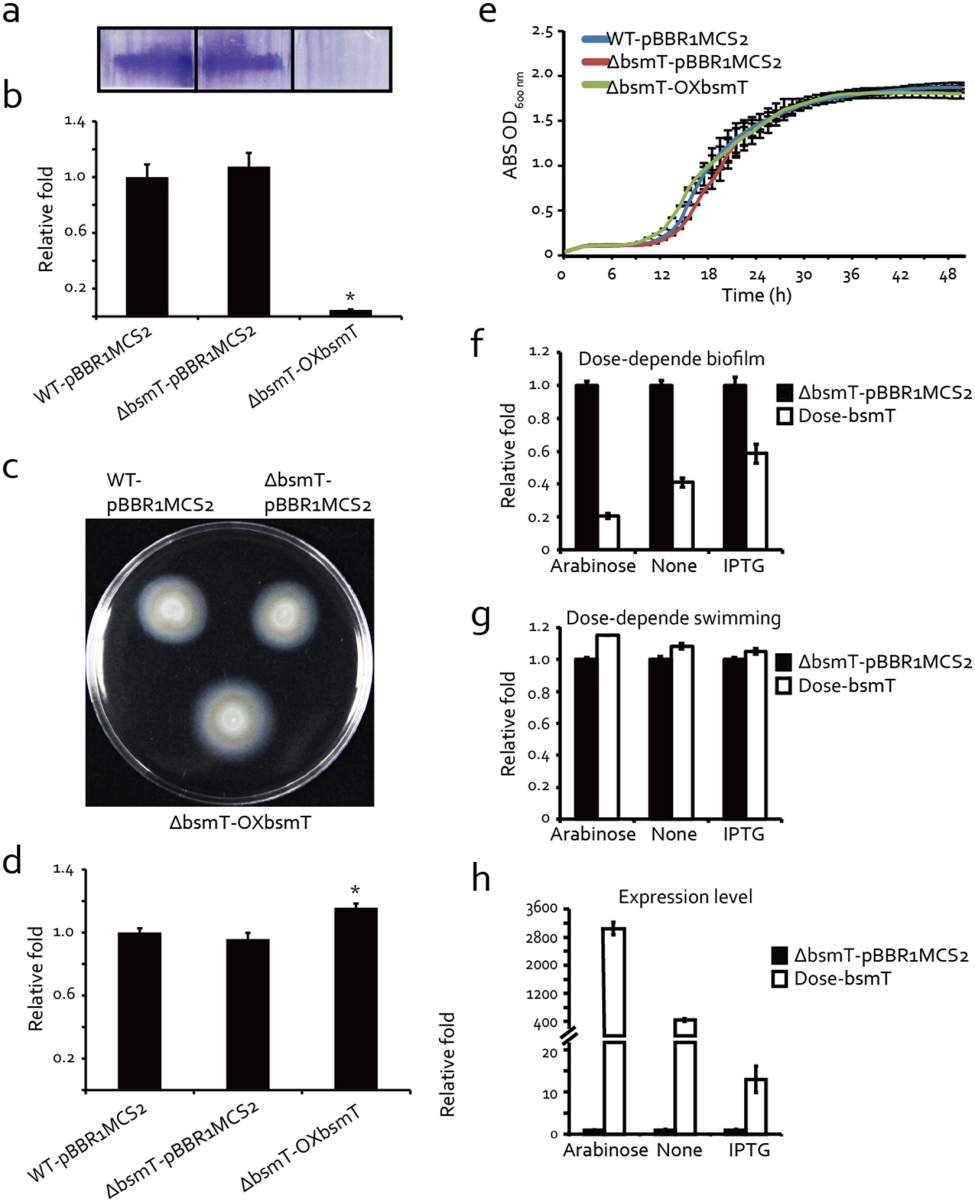
Phenotypic characterization shows that BsmT is a positive regulator of bacterial swimming motility and a negative regulator of biofilm formation. (**a**) Biofilm formation quantified by crystal violet staining. (**b**) Relative quantification of biofilm formation measured at 590 nm using a Tecan Infinite 200 Pro scanner. (**c**) Swimming motility analyses of bacterial strains on rich NYG medium plates containing 0.15% agar. (**d**) Relative quantification of bacterial swimming zone diameters. (**e**) Growth curves of bacterial strains grown on rich NYG medium at 28 °C. ABS: absorbance. (**f**) Dose-dependent effect on biofilm formation. Relative quantification of the biofilm was measured when 0.1% arabinose and 4 mM IPTG were used to suppress the expression of *bsmT*. (**g**) Dose-dependent effect on the swimming motility. Relative quantification of the diameters of the swimming zones was measured when 0.1% arabinose and 4 mM IPTG were used to suppress the expression of *bsmT*. (**h**) The expression level of *bsmT* along with the addition of 0.1% arabinose and 4 mM IPTG. Relative quantification of *bsmT* expression level using qRT-PCR assays. WT-pBBR1MCS2: wild-type strain containing a blank pBBR1MCS2 vector; ΔbsmT-pBBR1MCS2: blank pBBR1MCS2 vector-containing strain carrying an in-frame deletion of the gene *bsmT*; ΔbsmT-OXbsmT: the strain overexpressing *bsmT* in its in-frame deletion mutant; Dose-bsmT: *bsmT* in-frame deletion mutant containing the recombinant MpBBR1MCS2-bsmT. All data are representatives of at least triplicate repeatable experiments. The values are the means ± standard deviation (n = 3). *p < 0.05, as determined by ANOVA.

Since the *bsmR* operon contains four regulatory genes, we asked whether the other three genes are also involved in regulating bacterial swimming motility and biofilm formation, and whether there is a regulatory relationship between the four genes. In-frame deletion mutants and overexpressing strains of the other three genes were constructed and used in genetic analyses. As shown in Fig. 6a–d, the in-frame deletion of *bsmT* did not affect either bacterial swimming or biofilm production, but overexpressing *bsmT* significantly enhanced bacterial swimming motility, evidenced by the increase in the diameter of the swimming zone of strain ΔbsmT-OXbsmT to 115.6% of that of the WT strain. Moreover, in this strain bacterial biofilm formation was negligible, as it was only 4.6% of that measured in the WT strain. This pattern of phenotypic change resembled that of the *bsmR*-overexpressing strain. However, neither the deletion nor the overexpression of the other two HK genes, *DP16_RS18250* and *DP16_RS18240*, resulted in remarkable differences with the WT strain with respect to swimming motility and biofilm formation (Fig. S4). Again, the similar growth curves of all aforementioned recombinant strains ruled out the influence of bacterial growth on either of these activities (Fig. 6e and Fig. S4e,j). In addition, a dose-dependent assay was also carried out to exclude the potential artificial phenotypes caused by the overexpression of *bsmT*. As shown in Fig. 6f–h, the dose-dependent effects of biofilm quantities and swimming zones were also identified along with the decrease of the expression level of *bsmT*, which supported the view that *bsmT*, similar to *bsmR*, modulates biofilm formation and swimming motility in the bacterial cells. Thus, within the *bsmR* operon, only *bsmR* and *bsmT* are involved in the regulation of bacterial swimming motility and biofilm formation.

### *bsmT* activates the expression of *bsmR* in a positive feedback manner

**Figure 7:**
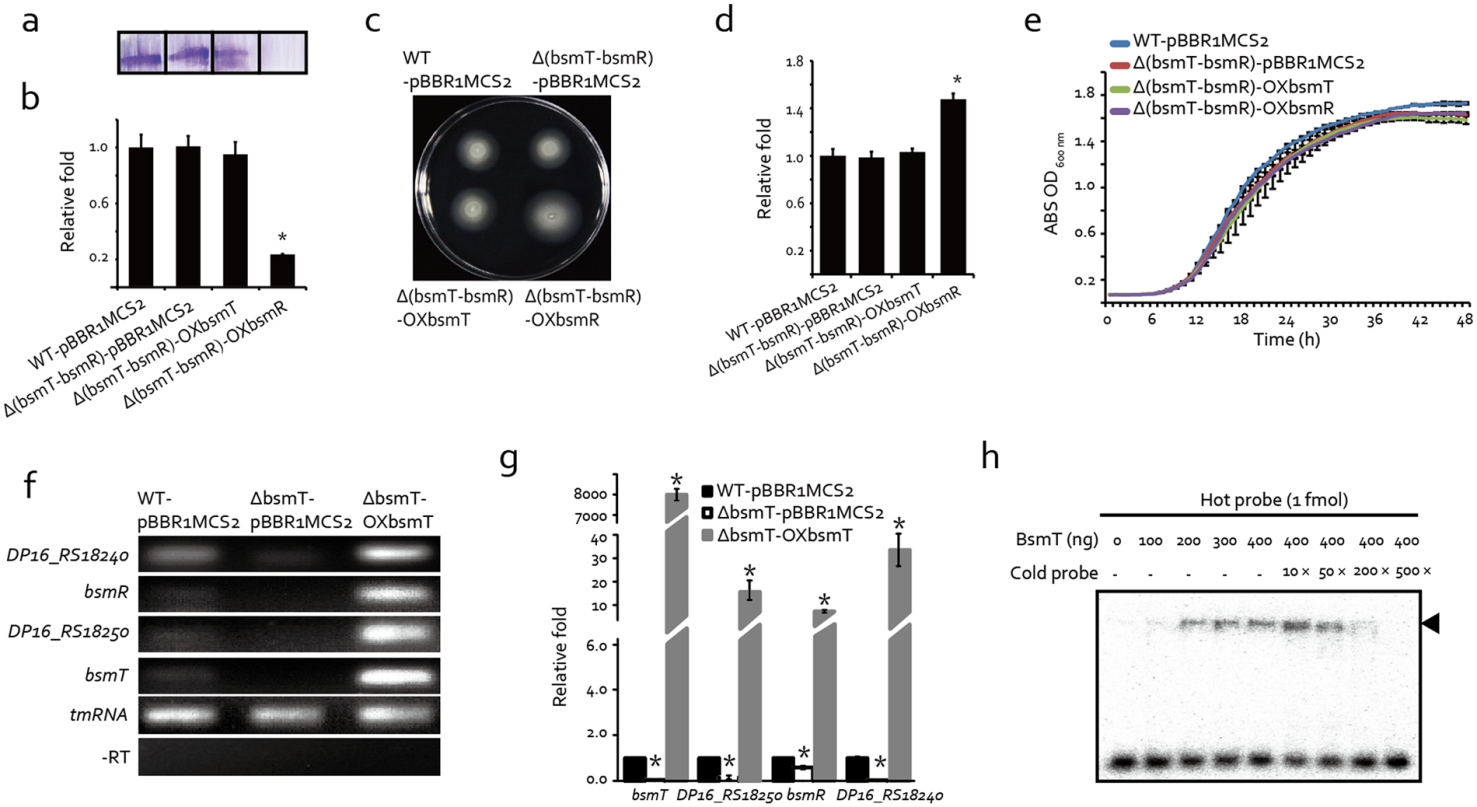
*bsmT* is epistatic to *bsmR* and regulates *bsmR* transcription through a positive feedback loop. (**a**) Biofilm levels of the bacterial strains quantified by crystal violet staining. (**b**) Relative quantification of biofilm formation. (**c**) Swimming motility analyses of bacterial strains grown on rich NYG plates containing 0.15% agar. (**d**) Relative quantification of the bacterial swimming zone diameters of the strains in (**c**). (**e**) Growth curves of bacterial strains grown in rich NYG medium at 28 °C. (**f**,**g**) *bsmT* positively regulates transcription of the *bsmR* operon. mRNA amounts were determined by semiquantitative RT-PCR (**f**) and quantitative RT-PCR (**g**), respectively. In (**f**), the bands represent PCR amplification products obtained using the cDNA generated from the total RNAs of the indicated strains. The tmRNA amplification product served as the loading control. −RT represents the negative control to detect the presence of contaminating DNA. (**h**) Electrophoretic mobility shift assay shows the direct binding of BsmT to the promoter region of the *bsmR* operon. 1 fmol of ^32^P-labeled double-stranded DNA corresponding to the promoter region of the *bsmR* operon was incubated at 28 °C in reaction buffer together with BsmT protein. Increasing amounts of unlabeled DNA served as the competitor. The black arrow indicates the position of the protein-DNA complex. (**b**,**d**,**e** and **g**) Each value is the average of three replicates. Error bars represent the standard deviations (n = 3). *p < 0.05, as determined by ANOVA. The qRT-PCR results (**g**) are representatives of triplicate repeatable experiments.

Since both *bsmR* and *bsmT* control bacterial swimming and biofilm formation, epistatic analysis was carried out to determine their regulatory relationship by constructing a double mutant of the two genes [strain Δ(bsmT-bsmR)-pBBR1MCS2] and then overexpressing *bsmR* and *bsmT*, respectively, in the resulting double mutant [strains Δ(bsmT-bsmR)-OXbsmR and Δ(bsmT-bsmR)-OXbsmT, respectively]. As shown in Fig. 7a–d, biofilm formation and swimming motility were similar in the double-mutant and WT strains. However, overexpressing *bsmR* against the double-mutant background caused a significant increase in the bacterial swimming zone, to 147.5% of that of the WT. It also sharply decreased biofilm formation, to 23.4% of that by the WT strain. The overexpression of *bsmT*, by contrast, did not cause a recognizable phenotypic difference with the double mutant and WT strains. There were no discrepancies in the growth of the tested strains (Fig. 7e). Accordingly, this experiment demonstrated that *bsmR* and *bsmT* function in the same signaling pathway to modulate bacterial swimming and biofilm formation, and that *bsmR* is in a regulatory position located downstream of *bsmT*.

Since the phosphorylation state of BsmR does not seem to regulate swimming or biofilm formation, and *bsmT* encodes a transcriptional regulator located in an operon together with *bsmR*, we proposed that the expression level of *bsmR* is critical in the regulation of swimming motility and biofilm formation and that *bsmT* positively regulates *bsmR* expression via a feedback loop. This hypothesis was verified by using semi-qRT-PCR and qRT-PCR to estimate the *bsmR* transcription level. As shown in Fig. 7f,g, in the WT and ΔbsmT mutant strains, all four genes of the *bfmR* operon were transcribed at extremely low levels. However, overexpressing *bsmT* significantly increased the mRNA levels of the four genes, by 6.3- to 198.6-fold compared to the WT levels. This result suggested that BsmT autoregulates the transcription of its own operon. Interestingly, the mRNA level of *DP16_RS18240* was higher than those of *bsmR, bsmT* and *DP16_RS18250* in the WT-pBBR1MCS2 strain, indicating that additional *cis*-regulatory elements drive *DP16_RS18240* expression. It was noteworthy that transcript level of *bsmR*, *bsmT* and *DP16_RS18250* was almost undetectable in the WT-pBBR1MCS2 strain (Fig. 7f), suggesting their expression remains a low level under the assay conditions. And to confirm the regulatory relationship between BsmT and the promoter region of the *bsmR* operon, possible protein-DNA interactions were examined in an electrophoretic mobility shift assay (EMSA). The results showed that recombinant BsmT-His_6_ formed stable protein-DNA complexes with the ^32^P-labeled DNA probe corresponding to the 5′ promoter sequence of the *bsmT-DP16_RS18250-bsmR-DP16_RS18240* operon. The addition of unlabeled probe resulted in effective competition with the labeled probe in binding to BsmT-His_6_ (Fig. 7h). Taken together, these results demonstrate that BsmT directly binds to the promoter region of its own operon and thereby regulates the transcription of all four genes (including *bsmR*) by a positive feedback loop.

## Discussion

The spatial and temporal control of c-di-GMP in bacterial cells is important to its modulation of diverse physiological processes and behaviors^50, 51^. However, the role of c-di-GMP in the nosocomial pathogen *S. maltophilia* is unclear. Our study provides genetic and biochemical evidence of c-di-GMP degradation by the BsmR protein, both *in vitro* and *in vivo*, by its EAL domain (Fig. 4a–c,h). As a regulatory protein of *S. maltophilia*, BsmR was shown to modulate the expression of 349 genes under the tested condition. These genes include the previously identified transcription factor FsnR, which is critical to the regulation of flagellar assembly (Fig. 5a,b, Supplementary Table S3). Although *bsmR* inactivation did not remarkably impact bacterial swimming or biofilm formation, the gradient increase in expression of the respective protein resulted in gradient increase in cellular swimming capability and decrease in biofilm development (Fig. 2f–h). We also determined that the transcription factor BsmT whose coding gene is located in the same tetra-cistronic operon as *bsmR*, binds directly to the promoter region of the operon and positively regulates *bsmR* transcription. Epistatic analysis confirmed that BsmR is located downstream of BsmT, where it regulates biofilm formation and bacterial swimming (Fig. 7). To our knowledge, BsmR is the first experimentally investigated protein involved in c-di-GMP turnover and the control of biofilm formation in *S. maltophilia*.

BsmR is a RR with an EAL domain as the output region. In general, RR phosphorylation is critical in regulating the biochemical activity of the corresponding output region, via a mechanism involving a conformational change^34^. For example, PleD, a RR with a GGDEF domain, controls flagellar motor activity in the gram-negative bacterium *Caulobacter crescentus*. The DGC activity of PleD is activated by phosphorylation-triggered dimerization^52, 53^. In *E. coli*, the RR CheB contains a methylesterase region responsible for the regulation of chemotaxis by the bacterium. The phosphorylation of CheB in response to a diverse range of chemoeffector concentrations activates its methylesterase region, which in turn modulates bacterial motility^54, 55^. However, RR regulation independent of phosphorylation has also been reported. For example, the RR VieA of *Vibrio cholerae* contains an EAL domain. VieA negatively regulates biofilm formation and virulence by decreasing the cellular c-di-GMP level. Its PDE activity is independent of the phosphorylation level because *in vitro* phosphorylation by acetyl phosphate did not affect the ability of the enzyme to degrade c-di-GMP, and neither a mutation of the conserved phosphorylation site nor the deletion of the REC-domain-coding sequence altered the control of downstream *vps* genes^56–58^. Subsequent analysis revealed that the PDE activity of VieA is activated by intracellular Mg^2+^ and Mn^2+^ but inhibited by Ca^2+^ and Zn^2+^ ^59, 60^. The results of our analysis suggest that, similar to VieA, the PDE activity of BsmR is independent of its phosphorylation state since no significant difference of the dose-dependent effects between BsmR and its phosphorylation site mutated proteins, was identified with respect to bacterial swimming motility and biofilm formation (Fig. 2f–h). The positive correlation between the *bsmR* transcription level and cellular c-di-GMP level revealed by the qRT-PCR assay and LC-MS/MS assay (Figs 2h and 4d) suggests that *bsmR* expression level is an important regulatory determinant of BsmR PDE activity.

The role of bacterial swimming in biofilm formation is complex and depends on the different stages of biofilm development. 1) In the early stage of cell aggregation, bacteria must move towards the liquid-solid inter-face, a process driven by flagellar activity (swimming). 2) However, after becoming fixed to the solid surface, where they continue to grow, bacteria no longer require motility, which instead must be inhibited to avoid dispersal of the biofilm. 3) When the biofilm reaches the mature stage, swimming motility is re-activated to release the bacterial cells so that they are able to colonize new habitats^8^. Our results showed that the overexpression of *bsmR* substantially increased swimming of *S. maltophilia*, while strongly reducing biofilm formation (Fig. 2). Thus, the abnormally increased swimming motility caused by the up-regulation of *bsmR* acted as a negative force hindering bacterial aggregation on a solid surface. This implies the need for the tight regulation of BsmR activity during biofilm formation. Besides the transcription factor BsmT that plays a positive role in activating *bsmR* transcription, inhibitory factors of BsmR are needed during the cellular aggregation stage of biofilm development. The identification of these inhibitors and of the mechanism underlying the integration of the positive and negative control of BsmR activity will require further studies.

As revealed by RNA-seq analysis, the overexpression of BsmR altered the expression of 349 genes, which suggests that BsmR is a global regulator of the physiological processes of *S. maltophilia* (Fig. 5a, Supplementary Table S3). Among these genes, BsmR modulates the transcriptions of 45 that are associated with signal transduction and cellular regulation, including 3 HKs, 2 RRs, 9 transcription factors, and 3 alternative sigma factors. This result places BsmR-triggered c-di-GMP degradation in the center of a sophisticated network that also serves to regulate biofilm development by *S. maltophilia*.

In the development of novel antibacterial agents, the targeting of biofilm formation is likely to be an effective therapeutic strategy, since disruption of this critical process, which involves cell-cell communication, adhesion to a solid surface, the assembly of an extracellular polysaccharide matrix, and, later, biofilm dispersal, will increase the susceptibility of bacteria to established antibiotics^61, 62^. The decreased cell aggregation achieved by activating BsmR PDE activity demonstrates the potential of chemical agonists of the BsmR PDE to block *S. maltophilia* biofilm development and thus nosocomial infections, given that these agonists are unlikely to harm human health and they do not activate the expression of bacterial virulence factors. An in-depth analysis of the BsmR regulatory cascade will allow the screening and identification of agonists able to control *S. maltophilia* biofilm formation and therefore infections by this bacterium.

## Methods

### Bacterial strains, plasmids, and culture conditions

The bacterial strains and plasmids used in this study are listed in Supplementary Table S1. *S. maltophilia* strains were cultured at 28 °C in rich NYG medium (5 g tryptone/l, 3 g yeast extract/l, 20 g glycerol/l, pH 7.0) or 210 medium (5 g sucrose/l, 8 g casein enzymatic hydrolysate/l, 4 g yeast extract/l, 3 g K_2_HPO_4_/l, 0.3 g MgSO_4_·7H_2_O/l, pH 7.0). If necessary, strains were grown to an OD_600 nm_ = 0.4 before the cells were harvested. *Escherichia coli* strain DH5α was used as the host to prepare all recombinant plasmids. *E. coli* strain BL21 (DE3) and pET30a (Novagen) vectors were used to express the His_6_-tagged recombinant proteins. In-frame deletion mutants were constructed by homologous, double-crossover recombination using the suicide vector pK18mobsacB. To construct genetically complementary strains, the recombinant broad-host-range vector pBBR1MCS2, carrying full-length sequences of the genes of interest (under the control of the Plac promoter), was electroporated into competent cells of *S. maltophilia* wild-type or mutant strains. Electroporation was performed in a Bio-Rad Pulser XCellTM (Bio-Rad, Hercules, CA, USA) at 18 kV/cm, 25 μF, and 200 Ω. Strains carrying point mutations were constructed using the Fast Mutagenesis System (Transgene Biotech, Beijing, China). All other general molecular biology operations were carried out according to standard molecular cloning protocols^63^. The antibiotics ampicillin (100 μg/ml), kanamycin (50 μg/ml), chloramphenicol (34 μg/ml) were added as needed. The primers used in the constructions are listed in Supplementary Table S2.

### Biofilm assays

*S. maltophilia* biofilm mass was quantified by crystal violet staining as previously reported^64, 65^. Briefly, *S. maltophilia* strains were cultured overnight at 28 °C in rich NYG medium with the addition of different concentrations of IPTG or arabinose, diluted to an OD_600 nm_ = 0.4, inoculated into 96-well polystyrene plates, and incubated at 28 °C for 6 h without shaking. The wells were washed rigorously with water, stained with 0.1% crystal violet for 20 min without shaking, washed rigorously with water, and dried. The crystal violet stain was solubilized in 95% ethanol and the released amount quantified using Tecan Infinite 200 Pro at 590 nm.

### Bacterial swimming motility assay

The assayed strains were cultured overnight at 28 °C in rich NYG medium with the addition of different concentrations of IPTG or arabinose, washed twice with 10 mM MgCl_2_, and diluted to an OD_600 nm_ = 0.4. Three microliters of the diluted cultures were then used to inoculate rich NYG medium plates containing 0.15% agar. The diameters of the swimming zones were measured after incubation of the plates at 28 °C for 24 h.

### RNA extraction and semi-quantitative and quantitative RT-PCR assays

Total bacterial RNA was extracted using TRIzol (Invitrogen) following the manufacturer’s instructions and then quantified using a NanoDrop spectrophotometer (Thermo Fisher). Contaminating DNAs in the total RNAs were removed using DNA-free DNase (Life Technology). The first strand of the cDNA was generated using random primers (Promega) and Superscript III reverse transcriptase (Invitrogen). cDNA of the transfer mRNA served as the loading control for semi-quantitative and quantitative RT-PCR. In the PCRs, samples lacking reverse transcriptase during first strand cDNA synthesis served as the negative control to evaluate potential DNA contamination, and the DNA template of the WT strain as the positive control. The primers used in the PCR are listed in Supplementary Table S2.

### High-throughput RNA sequencing assay

*S. maltophilia* strains were grown in rich NYG medium and incubated at 28 °C to OD_600 nm_ = 0.4. The cells were harvested by centrifugation and total RNA was then extracted. Three biological repeats of each sample were prepared. Contaminating DNA was removed using DNA-free DNase (Life Technology). mRNAs were enriched by removing ribosomal RNAs with the MICROBEnrich kit (Ambion, Austen, TX, USA) following the manufacturer’s instructions. High-throughput RNA sequencing was carried out on an Illumina HiSeq^TM^ 2000. Raw data were evaluated by the quality checks reported previously before further analysis^66^. (i) The distribution of nucleotide-level quality scores of more than 85% reads of both samples were more than 20, which means a high quantity of the sequencing run. (ii) The average and distribution of GC content of both samples were less than 60%, which would not induce biases in the quantification. (iii) 85.84% and 78.28% reads of ΔbsmR-OXbsmR and WT-pBBR1MCS2, respectively, were mapped to the gene sequences of *S. maltophilia* ATCC 13637, suggesting good quanlities of the samples. (iv) No significant decrease of the coverage from the 3′ to the 5′ ends of the transcripts was identified, suggesting no significant RNA degradation during sequencing. (v) rRNA Reads of ΔbsmR-OXbsmR and WT-pBBR1MCS2 were 1.78% and 5.25% of total reads, respectively, which would not affect the quantification. After the evaluation, all collected clean reads were aligned with the complete genomic sequence of *S. maltophilia* ATCC 13637 using SOAPaligner/SOAP2 using the standard of no more than 5 mismatches. Differentially expressed genes were screened by SOAPaligner/SOAP2 and the results were verified by Bowtle.

### Protein expression and purification

All recombinant proteins were expressed with a C-terminal His_6_-tag. Expression was achieved using pET30a (Novagen) vectors and *E. coli* BL21 (DE3) cells. The primers used in the expression vector constructions are listed in Supplementary Table S2. The proteins were expressed and purified according to the manufacturer’s instructions. In brief, the expression strains were inoculated in rich LB medium (10 g tryptone/L, 5 g yeast extract/L, 10 g NaCl/L) at 37 °C, grown to an OD_600 nm_ = 0.4–0.8, and induced at 16 °C for 16 h with 5 mM IPTG. The bacterial cultures were collected, sonicated, centrifuged, and used in Ni-nitrilotriacetic acid (Ni-NTA) affinity chromatography to collect the His_6_-tagged recombinant proteins according to the mannual. Then using Centricon YM-10 columns the proteins were stored in storage buffer (Tris-HCl 50 mM, EDTA 0.5 mM, NaCl 50 mM, glycerol 5%, pH 8.0) until further use.

### *In vitro* phosphroylation assay

*In vitro* phosphorylation was assayed as described previously^67^. In brief, the purified C-terminal-His_6_-tagged membrane proteins DP16_RS18240 and DP16_RS18250 were incubated at 28 °C with 100 μM ATP containing 10 μCi [γ-^32^P]-ATP (PerkinElmer, USA) in 20 μl of reaction buffer (50 mM Tris-HCl, pH 7.8; 2 mM DTT; 25 mM NaCl; 25 mM KCl; 5 mM MgCl2) using SreS as the control to prove the assay was working. To examine whether there was phosphotransfer from the two kinases to BsmR and BsmT, purified BsmR and BsmT proteins were added into the reaction system, respectively. Then aliquots of the samples for electrophoresis were retrieved at the indicated time points. The reactions in those samples were immediately stopped by the addition of 6 × SDS loading buffer [250 mM Tris-HCl pH 6.8, 10% (w/v) SDS, 0.5% (w/v) bromophenol blue, 50% (v/v) glycerol, 25 mM PMSF]. SDS-PAGE was carried out on 12% acrylamide gels, after which the gels were placed into Ziploc bags, exposed to phosphor screens for 1 h at room temperature, and scanned at 25-μM resolution using the PhosphorImage system Typhoon FLA7000 (Amersham Biosciences, Bath, UK). Protein locations and amounts were determined by staining the gels with Coomassie bright blue after autoradiography.

### *In vivo* phosphorylation assay

The *in vivo* phosphorylation assay was carried out following the Phos-tag Acrylamide Kit (Nard, USA). Briefly, *S. maltophilia* strains were grown in rich NYG medium and incubated at 28 °C to OD_600 nm_ = 0.4. The cells were harvested by centrifugation and lysed with 1 M formic acid. 30 s of vortexing were required for the complete lysis of the cells. Each lysate was solubilized by the addition of 6 × SDS loading butter and neutralized to pH 6.0 by the addition of 5 N NaOH. Samples, together with the purified unphosphorylated BsmR and BsmT proteins, were loaded on Phos-tag acrylmnide gels and run at 4 °C. After the separation samples were transferred to nitrocellulose membranes by a Bio-Rad semidry transfer. And the monoclonal antibody of His_6_ tag conjugated with HRP and Clarity^TM^ western ECL substrates (Bio-Rad) were used to image BsmR and BsmT proteins.

### *In vitro* phosphodiesterase assays

The method was described in previous studies^68–70^. The ^32^P-labeled c-di-GMP used in this assay was generated by incubating diguanylate cyclase tDGC^R158A^ with [α-^32^P]-GTP (PerkinElmer, USA) at 45 °C in 20 μl of c-di-GMP synthesis buffer (50 mM Tris-Cl, pH 8.0; 20 mM MgCl_2_; 250 mM NaCl; 1 mM DTT). The reaction was stopped by heating at 95 °C for 10 min. PDE assays were carried out by incubating the purified proteins with the synthesized c-di-GMP at 28 °C in reaction buffer (250 mM NaCl; 25 mM Tris, pH 8.0; 10 mM MgCl_2_; 5 mM β-mercaptoethanol). Aliquots of the samples were chronologically retrieved at the indicated time points and mixed with an equal volume of 0.5 M EDTA, pH 8.0, to stop the reactions. The c-di-GMP in the samples was separated by thin-layer chromatography on a polygram CEL 300 PEI cellulose thin layer chromatography plate. The plates were then placed into a Ziploc bag and exposed to a phosphor screen for 1 h before they were scanned at 25-μM resolution using the PhosphorImage system Typhoon FLA7000 (Amersham Biosciences, Bath, UK).

### Cellular c-di-GMP quantification by LC-MS/MS

*S. maltophilia* strains were grown in rich NYG medium and incubated at 28 °C to OD_600 nm_ = 0.4. The cells were harvested by centrifugation and resuspended in lysis buffer (40% acetonitrile, 40% methyl alcohol, 20% H_2_O). 30 s vortex and 15 min incubation on ice were needed to ensure the complete lysis. After that a 10-minute heating at 95 °C was carried out to inactivate all possible enzymatic activities of the lysate. Then the suspension were collected by centrifugation and evaporated to dryness by a vacuum freeze drier ZLS-1 (HeXi, Hunan, China). The pellet was solubilized using water and the c-di-GMP level was quantified by reverse phase-coupled HPLC-MS/MS. To calculate the c-di-GMP concentration in bacterial cells, bacterial cells used to extract c-di-GMP were numbered by diluting the bacterial culture and plated them on NYG plates for counting.

### *In vivo* FRET-based c-di-GMP biosensor assay

This method was previously described^43^. The FRET-based c-di-GMP biosensor reporting system was constructed by inserting the complete coding sequence of the fused YPet-YcgR-CyPet protein into the broad-host vector pBBR1MCS1, as previously reported. The recombinant vector was used to transform *S. maltophilia* strains by electroporation. The primers used in the constructions are listed in Supplementary Table S2. The recombinant protein Ypet-YcgR-CyPet was expressed, purified, and incubated with increasing concentrations of c-di-GMP (0–1,000 nM) in PBS buffer (pH 7.4). FRET values (528 nm/496 nm) were measured at 28 °C using a Synergy H4 (BioTech, USA) system with excitation at 425 nm and emission scanning from 460 to 560 nm in 2-nm intervals. *S. maltophilia* strains containing the FRET-based c-di-GMP biosensor system were grown in rich NYG medium to an OD_600 nm_ = 0.4 and used in FRET value measurements. The constructed table of FRET values and c-di-GMP concentrations was used to roughly determine the c-di-GMP concentrations of the *S. maltophilia* strains.

## Electronic supplementary material

All supplementary information files
