# Author Correction: BsmR degrades c-di-GMP to modulate biofilm formation of nosocomial pathogen *Stenotrophomonas maltophilia*

**DOI:** 10.1038/s41598-019-43380-7

**Published:** 2019-10-09

**Authors:** Wei Liu, Xiu-Qi Tian, Jin-Wei Wei, Li-Li Ding, Wei Qian, Zhong Liu, Fang-Fang Wang

**Affiliations:** 1 School of Pharmacy, Shanghai Jiao Tong University, Shanghai, 200240 China; 2 State Key Laboratory of Plant Genomics, Institute of Microbiology, Chinese Academy of Sciences, Beijing, 100101 China; 3 School of Life Sciences, University of Chinese Academy of Sciences, Beijing, 100049 China

Correction to: *Scientific Reports* 10.1038/s41598-017-04763-w, published online 05 July 2017

This Article contains typographical errors in the Acknowledgements section.

“The work was financially supported by National Natural Science Foundation of China (grant 3140071), and the Strategic Priority Research Program of the Chinese Academy of Sciences (grant XDB11040700).”

should read:

“The work was financially supported by National Natural Science Foundation of China (grant 31400071), and the Strategic Priority Research Program of the Chinese Academy of Sciences (grant XDB11040700).”

