# Supplementary material for: BsmR degrades c-di-GMP to modulate biofilm formation of nosocomial pathogen Stenotrophomonas maltophilia
Source: Sci Rep. 2017 Jul 5;7:4665. doi: 10.1038/s41598-017-04763-w (PMC5498567; doi:10.1038/s41598-017-04763-w)
Supplement: Supplementary file 1 — All supplementary information files [file 41598_2017_4763_MOESM1_ESM.pdf]

BsmR degrades c-di-GMP to modulate biofilm formation of nosocomial pathogen *Stenotrophomonas maltophilia*

Wei Liu[1,2], Xiu-Qi Tian[2,3], Jin-Wei Wei[2,3], Li-Li Ding[2,3], Wei Qian[2], Zhong Liu[1,*] & Fang-Fang Wang[2,*]

[1]School of Pharmacy, Shanghai Jiao Tong University, Shanghai, 200240, China.

[2]State Key Laboratory of Plant Genomics, Institute of Microbiology, Chinese Academy of Sciences, Beijing, 100101, China.

[3]School of Life Sciences, University of Chinese Academy of Sciences, Beijing, 100049, China.

*Correspondence authors

Correspondence and requests for materials should be addressed to F.F.W. (e-mail: wangff@im.ac.cn) or Z.L. (e-mail: liuzhong@sjtu.edu.cn)

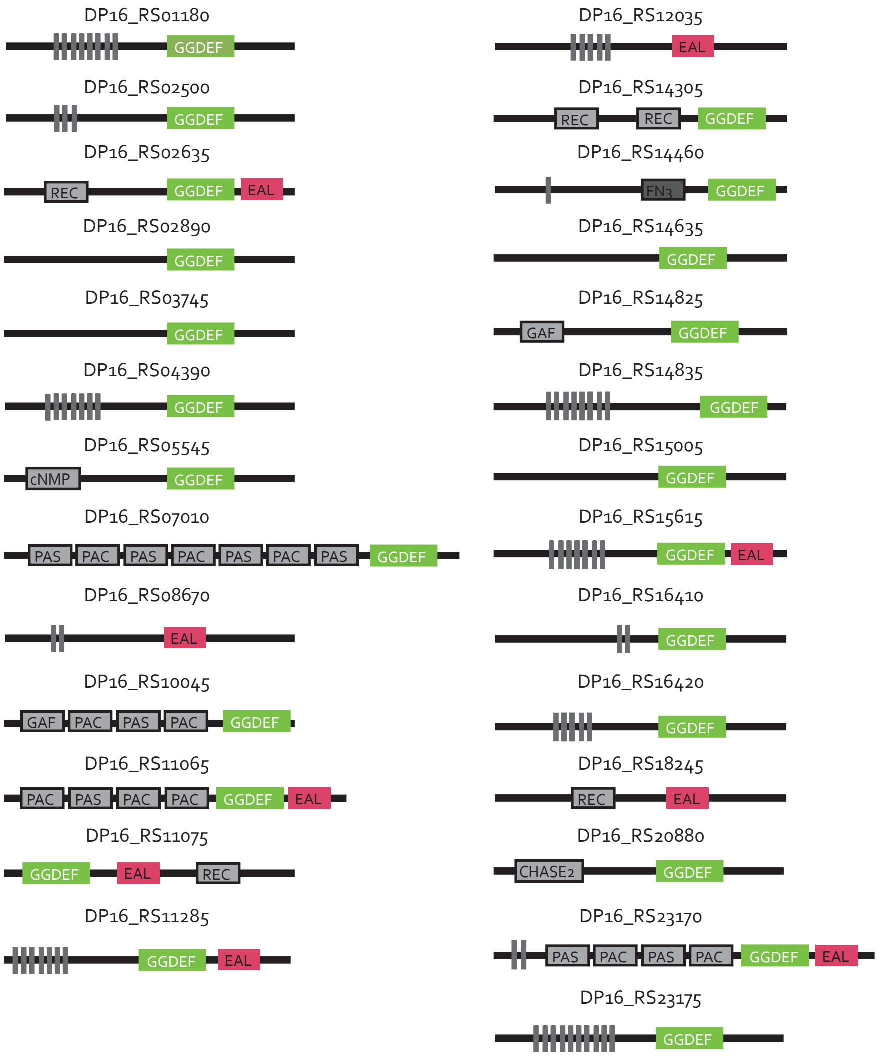

Figure S1. Putative secondary structures of proteins predicted to be involved in c-di-GMP metabolism in *S. maltophilia* CGMCC 1.1788. The Pfam database was searched using HMMer (E value ≤ 1.0). Domain names are according to the Pfam database. REC: response regulator receiver domain, PAS: PAS domain, cNMP: cyclic nucleotide-binding domain, GGDEF: GGDEF domain of diguanylate cyclase, EAL: EAL domain, PAC: PAS fold, GAF: GAF domain, CHASE2: CHASE2 domain, FN3: fibronectin type III domain. Gray vertical bars represent the predicted transmembrane helices. Protein names are listed.

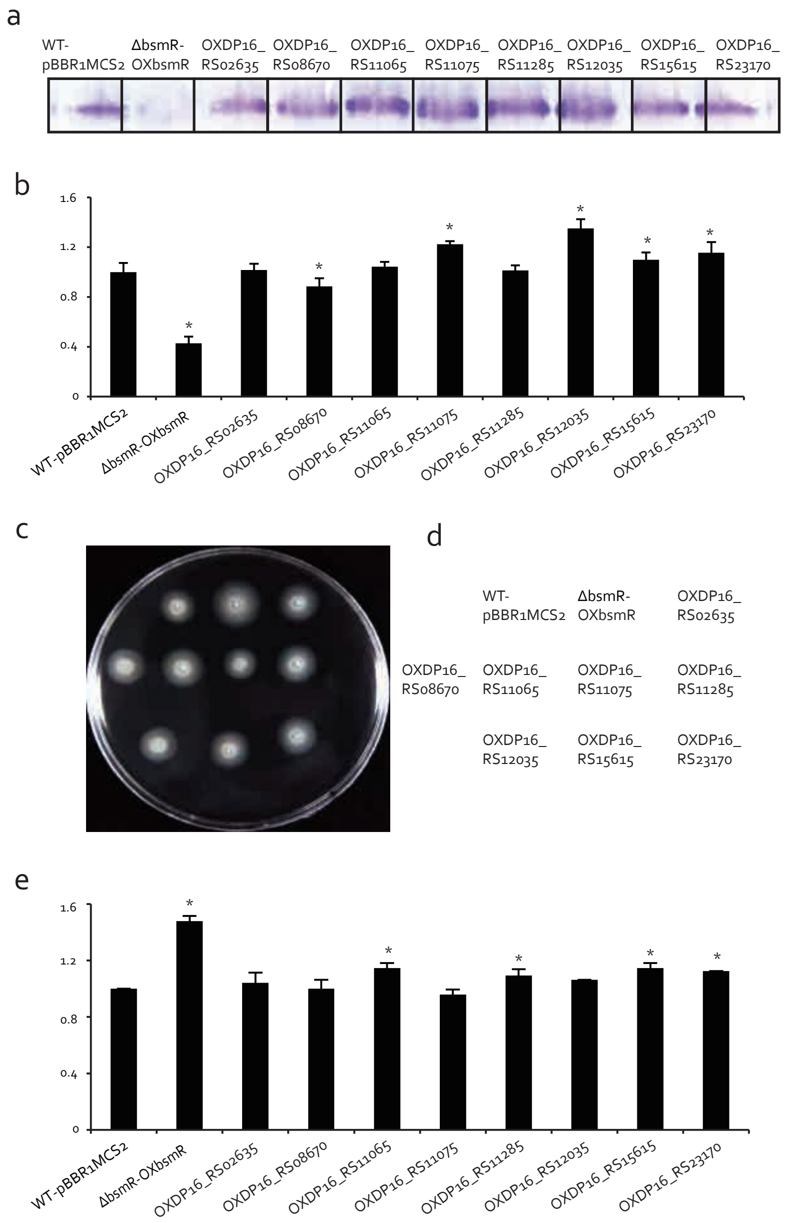

Figure S2. *bsmR* has specificity in controlling bacterial swimming motility and biofilm formation. (a) Biofilms formed by bacterial strains and evaluated by crystal violet staining. (b) Relative quantification of the biofilm in (a) at 590 nm using a Tecan Infinite 200 Pro scanner. The experiment was repeated 4 times. (c) Swimming motility of bacterial strains in rich NYG medium containing 0.15% agar. (d) The corresponding bacterial strains used in (c). (e) Relative quantification of the diameters of the bacterial swimming zones. The data are the average of three separate measurements. WT-pBBR1MCS2: wild-type strain containing the blank pBBR1MCS2 vector; ΔbsmR-OXbsmR: *bsmR* in-frame deletion mutant containing the recombinant pBBR1MCS2-bsmR vector; OXDP16_RS02635, OXDP16_RS08670, OXDP16_RS11065, OXDP16_RS11075, OXDP16_RS11285, OXDP16_RS12035, OXDP16_RS15615, OXDP16_RS23170: wild type strains containing the recombinant vectors pBBR1MCS2- DP16_RS02635, pBBR1MCS2-DP16_RS08670, pBBR1MCS2-DP16_RS11065, pBBR1MCS2-DP16_RS11075, pBBR1MCS2-DP16_RS11285, pBBR1MCS2-DP16_RS12035, pBBR1MCS2- DP16_RS15615, pBBR1MCS2-DP16_RS23170, respectively. All data are representatives of at least triplicate repeatable experiments. All values are the means ± standard deviations. * $p < 0.05$, as determined by ANOVA.

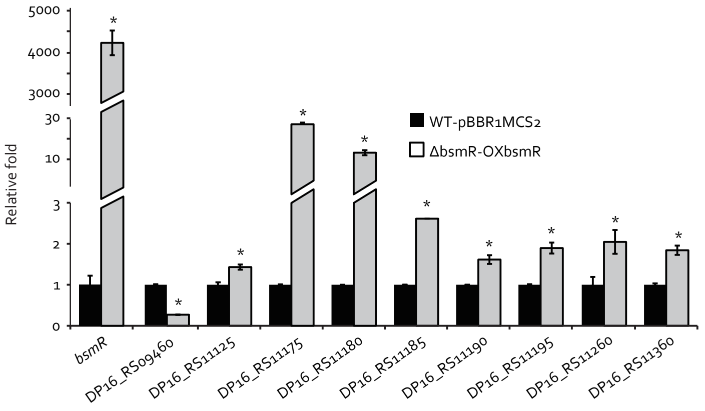

Figure S3 Verification of RNA-seq results by qRT-PCR. Relative quantification of 10 genes randomly selected from the RNA-seq results, including *bsmR*. Expression of the tmRNA was used as the loading control. Each value is the average of three replicates. Error bars represent the standard deviations (n = 3). *$p < 0.05$, as determined by ANOVA. The qRT-PCR results are representatives of triplicate repeatable experiments.

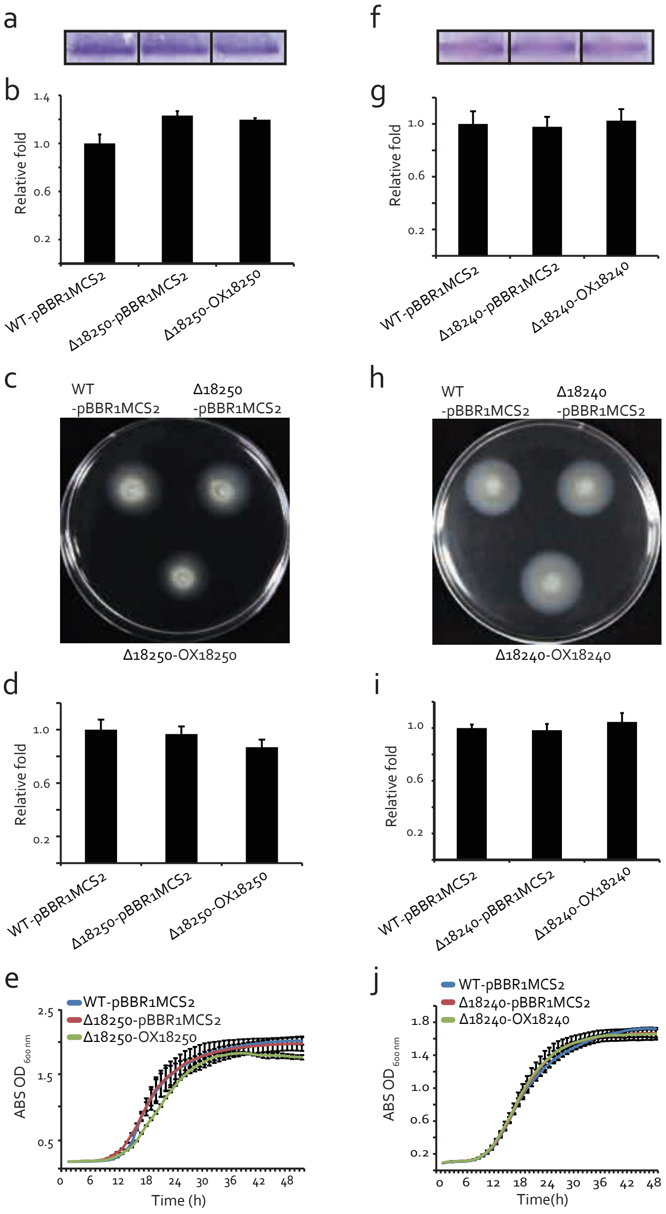

Figure S4. *DP16_RS18240* and *DP16_RS18250* are not involved in controlling bacterial swimming motility and bioiflm formation. (a,f) Biofilm formation quantified by crystal violet staining. (b, g) Relative quantification of biofilm formation measured at 590 nm using a Tecan Infinite 200 Pro scanner. (c, h) Swimming motility analyses of bacterial strains on rich NYG medium plates containing 0.15% agar. (d,i) Relative quantification of bacterial swimming zone diameters. (e, j) Growth curves of bacterial strains grown on rich NYG medium at 28 °C. ABS: absorbance. WT-pBBR1MCS2: wild-type strain containing a blank pBBR1MCS2 vector; Δ18240-pBBR1MCS2 and Δ18250-pBBR1MCS2: blank pBBR1MCS2 vector-containing strains carrying the in-frame deletion of *DP16_RS18240 and DP16_RS18250, respectively*; Δ 18240-OX18240 and Δ18250-OX18250: strains overexpressing *DP16_RS18240 and DP16_RS18250* in their in-frame deletion mutants, respectively;. All data are representatives of at least triplicate repeatable experiments. The values are the means ± standard deviation (n = 3). * $p < 0.05$, as determined by ANOVA.

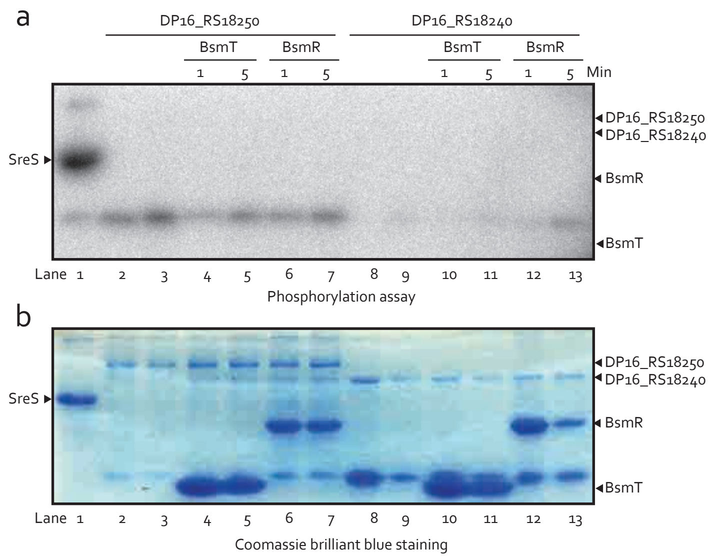

Figure S5. No kinase activities of DP16_RS18240 and DP16_RS18250 were detected. Membranes containing purified full-length histidine kinase were incubated at 28°C in reaction buffer containing [γ-^{32}P] ATP. The reactions were stopped by the addition of SDS-loading buffer at the indicated time points. Proteins in the samples were separated by SDS-PAGE on 12% acrylamide gels and then subjected to autoradiography. Black arrows indicate the positions of the protein bands of DP16_RS18240 and DP16_RS18250. (a) and (c) are autoradiography images of the gels. (b) and (d) are coomassie brilliant blue staining images of the gels in (a) and (c), respectively. The experiments were repeated three times.

Table S1. Bacterial strains and plasmids used in this study.

Strain and plasmid	Genotype or description	Resource or reference
**Strains**		
S. maltophilia ATCC 13637	Wild-type strain	CGMCC 1.1788
E. coli DH5α	Host strain used for molecular cloning	Lab collection
E. coli BL21(DE3)	Host strain used for protein expression	Lab collection
SM064	WT, Wild-type strain containing blank pBBR1MCS2 vector	This study
SM065	ΔbsmT-pBBR1MCS2, in-frame deletion mutant of *DP16_RS18255* containing blank pBBR1MCS2 vector	This study
SM066	Δ18250-pBBR1MCS2, in-frame deletion mutant of *DP16_RS18250* containing blank pBBR1MCS2 vector	This study
SM067	ΔbsmR-pBBR1MCS2, in-frame deletion mutant of *DP16_RS18245* containing blank pBBR1MCS2 vector	This study
SM068	Δ18240-pBBR1MCS2, in-frame deletion mutant of *DP16_RS18240* containing blank pBBR1MCS2 vector	This study
SM069	ΔbsmT-OXbsmT, complementary strain with *bsmT* overexpressed in ΔbsmT mutant	This study
SM070	Δ18250-OX18250, complementary strain with *DP16_RS18250* overexpressed in Δ18250 mutant	This study
SM071	ΔbsmR-OXbsmR, complementary strain with *bsmR* overexpressed in ΔbsmR mutant	This study
SM072	Δ18240-OX18240, complementary strain with *DP16_RS18240* overexpressed in Δ18240 mutant	This study
SM073	Δ(bsmT-bsmR)-pBBR1MCS2, double in-frame deletion mutant of *DP16_RS18245* and *DP16_RS18255* containing blank pBBR1MCS2 vector	This study
SM074	Δ(bsmT-bsmR)-OXbsmT, epistasis analysis strain with *bsmT* overexpressed in Δ(bsmT-bsmR) mutant	This study
SM075	Δ(bsmT-bsmR)-OXbsmR, epistasis analysis strain with *bsmR* overexpressed in Δ(bsmT-bsmR) mutant	This study
SM076	ΔbsmR-OXbsmR^{D54A}, complementary strain with *bsmR*^{D54A} overexpressed in ΔbsmR mutant	This study
SM077	ΔbsmR-OXbsmR^{D54E}, complementary strain with *bsmR*^{D54E} overexpressed in ΔbsmR mutant	This study
SM078	ΔbsmR-OXbsmR^{E117A}, complementary strain with *bsmR*^{E117A} overexpressed in ΔbsmR mutant	This study
SM079	SM064 containing the pBBR-FRET plasmid	This study
SM080	SM067 containing the pBBR-FRET plasmid	This study
SM081	SM071 containing the pBBR-FRET plasmid	This study
SM082	SM078 containing the pBBR-FRET plasmid	This study
SM083	Dose-bsmR, *bsmR* in-frame deletion mutant containing the recombinant vector MpBBR1MCS2::*bsmR*	This study
SM084	Dose-bsmR^{D54A}, *bsmR* in-frame deletion mutant containing the recombinant vector MpBBR1MCS2::*bsmR*^{D54A}	This study
SM085	Dose-bsmR^{D54E}, *bsmR* in-frame deletion mutant containing the recombinant	This study

	vector MpBBR1MCS2::*bsmR*^{D54E}	
SM086	Dose-bsmR^{E117A}, *bsmR* in-frame deletion mutant containing the recombinant vector MpBBR1MCS2::*bsmR*^{E117A}	This study
SM087	Dose-bsmT, *bsmR* in-frame deletion mutant containing the recombinant vector MpBBR1MCS2::*bsmT*	This study
SM088	OXDP16_RS02635, overexpressing *DP16_RS02635* in WT strain	This study
SM089	OXDP16_RS08670, overexpressing *DP16_RS08670* in WT strain	This study
SM090	OXDP16_RS11065, overexpressing *DP16_RS11065* in WT strain	This study
SM091	OXDP16_RS11075, overexpressing *DP16_RS11075* in WT strain	This study
SM092	OXDP16_RS11285, overexpressing *DP16_RS11285* in WT strain	This study
SM093	OXDP16_RS12035, overexpressing *DP16_RS12035* in WT strain	This study
SM094	OXDP16_RS15615, overexpressing *DP16_RS15615* in WT strain	This study
SM095	OXDP16_RS23170, overexpressing *DP16_RS23170* in WT strain	This study
**Plasmids**		
pK18mob	Suicide vector to create a mutant by a single crossover; Kan^r	Schäfer *et al.*
pK18mobsacB	Suicide vector to create a mutant by a double crossover; Kan^r	Schäfer *et al.*
pET30a	Protein expression vector; Kan^r	Novagen
pBBR1MCS1	Broad-host-range vector used for genetic complementation; Chlo^r	Lab collection
pBBR1MCS2	Broad-host-range vector used for genetic complementation; Kan^r	Lab collection
pBBR-bsmT	pBBR1MCS2::*bsmT*, genetic complementation vector	This study
pBBR-18250	pBBR1MCS2::*18250*, genetic complementation vector	This study
pBBR-bsmR	pBBR1MCS2::*bsmR*, genetic complementation vector	This study
pBBR-18240	pBBR1MCS2::*18240*, genetic complementation plasmid	This study
pBBR-bsmR^{D54A}	pBBR1MCS2::*bsmR*^{D54A}, genetic complementation plasmid	This study
pBBR-bsmR^{D54E}	pBBR1MCS2::*bsmR*^{D54E}, genetic complementation plasmid	This study
pBBR-bsmR^{E117A}	pBBR1MCS2::*bsmR*^{E117A}, genetic complementation plasmid	This study
pBBR-FRET	pBBR1MCS1::*cfp – ycgR - yfp*,genetic complementation plasmid	This study
MpBBR-bsmR	pBBR1MCS2::*bsmR* with the *lacZ* promoter substituted by the arabinose P_{BAD} promoter and its suppresser gene *araC*, dose expression plasmid	This study
MpBBR-bsmR^{D54A}	pBBR1MCS2::*bsmR*^{D54A} with the *lacZ* promoter substituted by the arabinose P_{BAD} promoter and its suppresser gene *araC*, dose expression plasmid	This study
MpBBR-bsmR^{D54E}	pBBR1MCS2::*bsmR*^{D54E} with the *lacZ* promoter substituted by the arabinose P_{BAD} promoter and its suppresser gene *araC*, dose expression plasmid	This study
MpBBR-bsmT	pBBR1MCS2::*bsmT* with the *lacZ* promoter substituted by the arabinose P_{BAD} promoter and its suppresser gene *araC*, dose expression plasmid	This study
pBBR-DP16_RS02635	pBBR1MCS2:: *DP16_RS02635*, overexpression plasmid	This study
pBBR-DP16_RS08670	pBBR1MCS2:: *DP16_RS08670*, overexpression plasmid	This study
pBBR-DP16_RS11065	pBBR1MCS2:: *DP16_RS11065*, overexpression plasmid	This study
pBBR-DP16_RS11075	pBBR1MCS2:: *DP16_RS11075*, overexpression plasmid	This study
pBBR-DP16_RS11285	pBBR1MCS2:: *DP16_RS11285*, overexpression plasmid	This study
pBBR-DP16_RS12035	pBBR1MCS2:: *DP16_RS12035*, overexpression plasmid	This study
pBBR-DP16_RS15615	pBBR1MCS2:: *DP16_RS15615*, overexpression plasmid	This study
pBBR-DP16_RS23170	pBBR1MCS2:: *DP16_RS23170*, overexpression plasmid	This study
pET30a- bsmT	pET30a:: *bsmT*, for BsmT expression	This study

pET30a-18250	pET30a::*18250*, for 18250 expression	This study
pET30a- bsmR	pET30a:: *bsmR*, for BsmR expression	This study
pET30a-18240	pET30a::*18240*,for 18240 expression	This study
pET30a-FRET	pET30a::*yfp – ycgR - cfp*, for FRET analysis	This study

Table S2 Primers used in this study.

Primer	Sequence (5'-3')	Purpose
ΔbsmT	AAGCTTATGACCACACGGATCCTC	Construction of *bsmT* in-frame deletion mutant
	GGTACCCTGTGGCCGGGTCCGGGTC	
	GGTACCGGCGGCATGAGCGTGGGC	
	GAATTCTCAGGCGAACAACGAGGC	
OXbsmT	AAGCTTATGACCACACGGATCCTC	For *bsmT* complementation
	GAATTCTCAGGCGAACAACGAGGC	
ProbsmT	CATATGACCACACGGATCCTCATC	For BsmT expression
	AAGCTTGGCGAACAACGAGGCCTG	
Δ18250	AAGCTTATGCCGGCCGAGGCCGCC	Construction of *18250* in-frame deletion mutant
	GGATCCGAACGTGCCGTAGAAGCG	
	GGATCCACCGATACGCGCGATGAT	
	GAATTCTCATGGCGGGTTCGGGTC	
OX18250	AAGCTTATGCCGGCCGAGGCCGCC	For *18250* complementation
	GAATTCTCATGGCGGGTTCGGGTC	
Pro18250	CATATGCCGGCCGAGGCCGCCCCTGT	For 18250 expression
	AAGCTTTGGCGGGTTCGGGTCGTCAG	
ΔbsmR	AAGCTTATGACCCAGCGTGTCCTG	Construction of *bsmR* in-frame deletion mutant
	GGATCCGGCCGCCACCGGCTTGGG	
	GGATCCGCGCTGGCCAACCAGCTGG	
	GAATTCTCAGCTCAGGGCGGCTCG	
OXbsmR	AAGCTTATGACCCAGCGTGTCCTG	For *bsmR* complementation
	GAATTCTCAGCTCAGGGCGGCTCG	
ProbsmR	CATATGACCCAGCGTGTCCTGATC	For BsmR expression
	AAGCTTGCTCAGGGCGGCTCGCGA	
D54A	TACGACCTGGTGGTCAGTGCCCTGCTGATGCCCG	Construction of BsmR^{D54A}
	GCACTGACCACCAGGTCGTAGGGCTGGCAGGCA	
D54E	TACGACCTGGTGGTCAGTGAGCTGCTGATGCCCG	Construction of BsmR^{D54E}
	CTCACTGACCACCAGGTCGTAGGGCTGGCAGGCA	
E177A	AGGGCGGGTCGTGGCGGCCGCGGCACTGGTGCGC	Construction of BsmR^{E117A}
	GCGGCCGCCACGACCCGCCCTGACTGCAGTGACT	
Δ18240	AAGCTTATGTTCCGCTTCCCCCGC	Construction of *18240* in-frame deletion mutant
	GGATCCCCCTTCGCTGTCCGCAGC	
	GGATCCGAGCTGGCACAGGATGGC	
	GAATTCTCATGCGGGATCTCCTTG	
OX18240	AAGCTTATGTTCCGCTTCCCCCGC	For *18240* complementation
	GAATTCTCATGCGGGATCTCCTTG	
Pro18240	CATATGTTCCGCTTCCCCCGCCCTTC	For 18240 expression
	AAGCTTTGCGGGATCTCCTTGGCGC	
P18240	GTGCCCGGCCAATGTCGG	P*18240* probe for EMSA
	GCAACGATGCTAGGAAGC	

OXfsnR	AAGCTTGTGCGAGTTCTCATCG	For *fsnR* complementation
	GAATTCTTACTCCAGCTCGTGC	
QbsmT	AAGCTTATGACCACACGGATCCTC	For RT-PCR,*bsmT*
	GGTACCCTGTGGCCGGGTCCGGGTC	
Q18250	AAGCTTATGCCGGCCGAGGCCGCC	For RT-PCR,*18250*
	AAGCGCTCCTGCTCGCGAAG	
QbsmR	AAGCTTATGACCCAGCGTGTCCTG	For RT-PCR,*bsmR*
	TGGATGAACTGGATGCCGTCC	
Q18240	AAGCTTATGTTCCGCTTCCCCCGC	For RT-PCR,*18240*
	ATAGATCTGGGCCCTGTAGGC	
S60T	ATTGCACGCCAGCTGCAGCGCAG	*18260-bsmT*, for operon structure analysis
	ACTGGCGCGGCAGGGCCGCGTCG	
ST50	CGATGACCTGGTCACCCAGGAGG	*bsmT-18250*, for operon structure analysis
	CACGACCTCGAGGTCCAGCTCC	
S50R	GCCTGCTGGACGAAGAAGCACCG	*18250-bsmR*, for operon structure analysis
	ACACGCAGTGAGCGGCGAGCGAG	
SR40	GCGCTGCCTGGCCAGTGGCATGC	*bsmR-18240*, for operon structure analysis
	ATCCACGTCTTCGCAGGCATCG	
S4035	GGATGCCACCTACACCGCGTATC	*18240-18235*, for operon structure analysis
	CGGTCAGGCAGCGCAACAGCACC	
FRET	CATATGCACCACCACCACCACCACTCTAAAGGTGAAGAA	For FRET biosensor protein expression
	ACTAGTTTTGTACAATTCATTCATACCC	
	ACTAGTAGTGGTTACAATGAGCAGTT	
	GGTACCTTCTCGCACTTTATTCGCT	
	GGTACCTCTAAAGGTGAAGAATTATTC	
	AAGCTTTTATTTGTACAATTCATCCATA	
OXFRET	AAGCTTATGTCTAAAGGTGAAG	For FRET recombinant vector construction
	GGATCCTTATTTGTACAATTCA	
pBAD-bsmR	AAGCTTGCGTTAATATTTTGTTAA	For
	GAGCTCGCGTATTGGGCGCATGCAT	MpBBR1MCS2::*bsmR*/*bsmR*^{D54A}/*bsmR*^{D54E}/*bsmR*^{E117A}
	CCGTTGCAGCCCTAGATCGGC	construction
	GTTCATCGGCAACAAAGCGCAG	
	GAGCTCACTCCAGAAAGGCCACCAAC	
	AAGCTTTCAATGGTGATGGTGATGATG	
	GAATTCAGGAGGACAGCTATGACCACACGGATC	
	AAGCTTTCAATGGTGATGGTGATGATGGGCGAACAACGAGGC	
pBAD-bsmT	GAATTCAGGAGGACAGCTATGACCACACGGATC	For MpBBR1MCS2::*bsmT* construction
	AAGCTTTCAATGGTGATGGTGATGATGGGCGAACAACGAGGC	
OX02635	AAGCTTATGGCCGCAAACGAATCC	For pBBR1MCS2::*DP16_RS02635* construction
	GAATTCTCAGAGCACCGCGTTGTG	
OX08670	AAGCTTATGAGCCGGCTGCGCGTG	For pBBR1MCS2::*DP16_RS08670* construction
	GAATTCCTACTCCACTCCCTCACG	
OX11065	AAGCTTGTGGACCAAGGGATCATC	For pBBR1MCS2::*DP16_RS11065* construction
	CAGGTCGCGCAGTACCAC	

	GACCTGTTCGGCCGCTTC	
	GAGCTCTCACCGCATCAGCATCAC	
OX11075	GGTACCATGTGGAATCCCAACCAG	For pBBR1MCS2::*DP16_RS11075* construction
	GAATTCTTACCGCAGCGGGCGATC	
OX11285	AAGCTTATGCTGGTAGGCACGTAC	For pBBR1MCS2::*DP16_RS11285* construction
	GAATTCCTACAGGGGGGCGTTGTC	
OX12035	AAGCTTATGGATGCGCTTTCACGG	For pBBR1MCS2::*DP16_RS12035* construction
	GAATTCCTAGCTGGCGGCGAAGCG	
OX15615	AAGCTTATGACTGGCAGTTACAGT	For pBBR1MCS2::*DP16_RS15615* construction
	GAATTCTCACCCCACCCGGCGCAG	
OX23170	AAGCTTATGCAGGACGGGGTCAGG TCGACCACGCCGGGCAGG	For pBBR1MCS2::*DP16_RS23170* construction
	CTGCTCGGCAAGCAGGCG	
	GAATTCTTAGGCGGCCACCTCATC	

Table S3. List of differentially expressed genes in ΔbsmR-OXbsmR.

GeneID	Definition	Fold change (ΔbsmR-OXbsmR/WT)
**Pilli, flagellar assembly and other cell component protein (66)**		
DP16_RS03485	cell surface protein	2.620753861
DP16_RS06900	lipoprotein	0.393844830
DP16_RS08500	flagellar motor protein MotA	2.503775451
DP16_RS08505	flagellar motor protein MotB	2.283598409
DP16_RS11085	flagella protein	2.041179397
DP16_RS11095	flagellar basal body P-ring biosynthesis protein FlgA	5.838669994
DP16_RS11105	flagellar biosynthesis protein FlgB	5.251264406
DP16_RS11110	flagellar basal-body rod protein FlgC	3.141381386
DP16_RS11115	flagellar basal body rod modification protein	4.316772157
DP16_RS11120	flagellar hook protein FlgE	6.021594516
DP16_RS11125	flagellar basal body rod protein FlgF	5.405713360
DP16_RS11130	flagellar basal body rod protein FlgG	5.894276375
DP16_RS11135	flagellar basal body L-ring protein	7.501806295
DP16_RS11140	flagellar biosynthesis protein FlgI	4.893005067
DP16_RS11145	flagellar rod assembly protein FlgJ	4.155605855
DP16_RS11150	flagellar hook protein FlgK	3.800257136
DP16_RS11155	flagellar hook protein FlgL	3.750903147
DP16_RS11160	flagellin	3.123928963
DP16_RS11165	flagellin	2.863606579
DP16_RS11170	flagellin	2.897257381
DP16_RS11175	flagellar hook protein FliD	2.429428088
DP16_RS11180	flagellar protein FliS	2.426500204
DP16_RS11185	flagellar FliT family protein	2.712050070
DP16_RS11215	flagellar hook-basal body protein	5.583730822
DP16_RS11220	flagellar MS-ring protein	4.826981919
DP16_RS11225	flagellar motor switch protein FliG	4.249440908
DP16_RS11230	flagellar assembly protein FliH	3.852829863
DP16_RS11235	flagellar protein FliI	4.537077292
DP16_RS11240	flagellar export protein FliJ	3.375812833
DP16_RS11245	flagellar hook-length control protein	3.663672842
DP16_RS11250	flagellar basal body protein FliL	4.584437180
DP16_RS11255	flagellar motor switch protein FliM	3.949480373
DP16_RS11260	flagellar motor switch protein FliN	3.084075921
DP16_RS11265	flagellar protein	4.782401513
DP16_RS11270	flagellar biosynthesis protein flip	4.002488115
DP16_RS11280	flagellar biosynthetic protein FliR	3.438327885
DP16_RS11295	flagellar biosynthesis protein FlhB	2.016995089
DP16_RS11300	flagellar biosynthesis protein FlhA	3.055882858
DP16_RS11305	flagellar biosynthesis regulator FlhF	4.604767279
DP16_RS11335	flagellar motor protein	2.856017625

DP16_RS11340	flagellar motor protein MotD	2.983672958
DP16_RS11345	chromosome partitioning protein ParA	2.708431395
DP16_RS14620	pilus assembly protein	2.280954617
DP16_RS17150	baseplate assembly protein	4.480245426
DP16_RS17155	phage baseplate protein	18.441940475
DP16_RS17160	baseplate assembly protein	10.056300680
DP16_RS17165	phage tail protein I	4.843199119
DP16_RS17170	tail protein	9.297551177
DP16_RS17180	tail protein	22.274243430
DP16_RS17185	phage major tail tube protein	15.838573907
DP16_RS17190	mu-like prophage FluMu gp41 family protein	14.824048077
DP16_RS17195	phage tail tape measure protein	12.262173814
DP16_RS17200	tail protein	8.265879158
DP16_RS17205	phage tail protein	3.783238519
DP16_RS17210	phage tail protein	4.174392212
DP16_RS17580	prophage tail length tape measure family protein	2.107125003
DP16_RS18975	outer membrane beta-barrel domain protein	2.238334807
DP16_RS19060	fimbrial protein	0.284304679
DP16_RS19065	putative outer membrane usher protein mrkc precursor	0.331418589
DP16_RS19900	phage portal protein	4.606301831
DP16_RS20000	bacteriophage lambda head decoration family protein	3.651614535
DP16_RS20005	minor capsid protein E	3.824424977
DP16_RS20040	tail protein	3.479390051
DP16_RS20055	tail protein	3.735009490
DP16_RS20070	tail assembly protein	3.545261527
DP16_RS22795	phage Tail Collar domain protein	2.099690349
**Transmembrane and membrane protein with unknown function (11)**		
DP16_RS00845	putative transmembrane protein	2.484973335
DP16_RS02830	putative transmembrane protein	6.000955362
DP16_RS05180	prokaryotic cytochrome b561 family protein	2.031739205
DP16_RS08820	membrane protein	2.127377905
DP16_RS10635	membrane protein	0.315777058
DP16_RS11060	membrane protein	2.035291196
DP16_RS17075	membrane protein	2.574052344
DP16_RS20085	membrane protein [Proteobacteria]	3.081099014
DP16_RS20090	membrane protein	3.511483798
DP16_RS21320	membrane protein	2.525809781
DP16_RS23075	membrane protein	2.453918780
**Transposase and restriction-modification system (2)**		
DP16_RS20125	eco57I restriction-modification methylase family protein	2.854714207
DP16_RS22100	transposase	2.883008004
**Secrection system (3)**		
DP16_RS03490	two-partner secretion systemprotein	2.248137463
DP16_RS07195	type VI secretion protein	2.194219672

DP16_RS10575	type II secretory pathway, component ExeA	2.615761405
**Transport protein (22)**		
DP16_RS00865	ion channel	2.462942428
DP16_RS01390	TonB-dependent receptor	2.547517710
DP16_RS06080	TonB-dependent receptor	2.921691992
DP16_RS07245	MFS transporter	0.416589669
DP16_RS09395	nitrate/nitrite transporter NarK	0.316678136
DP16_RS09425	major facilitator transporter	0.286289936
DP16_RS09440	molybdate ABC transporter substrate-binding protein	0.487259423
DP16_RS10200	MFS transporter	2.994672674
DP16_RS10345	ABC transporter permease	3.811013775
DP16_RS10350	porin	3.053060701
DP16_RS10370	MFS transporter	2.679216534
DP16_RS10415	PTS fructose transporter subunit IIA	0.327352716
DP16_RS10630	iron permease	0.454627095
DP16_RS10640	cation transporter	0.455655488
DP16_RS10650	cation transporter	0.472860506
DP16_RS11720	major facilitator transporter	2.210819861
DP16_RS13390	TonB-dependent receptor	2.513105109
DP16_RS15745	peptide transporter	0.404927044
DP16_RS17080	TonB-dependent receptor	3.408569970
DP16_RS17360	membrane transporter	2.374241886
DP16_RS17420	sulfate transporter	2.457488269
DP16_RS21995	ligand-gated channel	0.492474694
**Metabolism (75)**		
DP16_RS00300	cysteine synthase A	0.459541564
DP16_RS01000	alpha-1,2-mannosidase family protein	2.348126361
DP16_RS02850	pyruvate dehydrogenase (acetyl-transferring) E1 component subunit alpha	2.225293009
DP16_RS02855	2-oxoisovalerate dehydrogenase subunit beta	2.037352409
DP16_RS02860	variant SH3 domain protein	2.068334368
DP16_RS02865	branched-chain alpha-keto acid dehydrogenase subunit E2	2.717516743
DP16_RS02890	diguanylate cyclase	2.449191693
DP16_RS03870	2,5-didehydrogluconate reductase B	0.337449596
DP16_RS04390	diguanylate cyclase	3.034711284
DP16_RS04950	glutamate synthase large subunit	0.491647582
DP16_RS05700	enoyl-CoA hydratase	2.252260234
DP16_RS06770	L-lactate permease	0.387369187
DP16_RS08320	phasin family protein	0.413588867
DP16_RS09260	diacylglycerol kinase	2.332878787
DP16_RS09365	molybdopterin-guanine dinucleotide biosynthesis protein MoaC	0.296919319
DP16_RS09370	molybdopterin molybdenumtransferase MoeA	0.355051605
DP16_RS09375	putative molybdopterin converting factor subunit 1	0.491189698
DP16_RS09420	rotamase	0.298333224

DP16_RS09470	glycogen-branching enzyme	0.498631966
DP16_RS09880	L1 family subclass B3 metallo-beta-lactamase	0.351400400
DP16_RS10150	glyoxalase	0.456840768
DP16_RS10185	2-keto-3-deoxygluconate kinase	3.321112162
DP16_RS10190	lipase	3.798991649
DP16_RS10265	PHB depolymerase esterase	2.405470497
DP16_RS10330	4-oxalomesaconate tautomerase	2.415946298
DP16_RS10335	nitrate ABC transporter ATPase	3.131188714
DP16_RS10340	mannosyltransferase	2.939897061
DP16_RS10410	1-phosphofructokinase	0.498132308
DP16_RS11310	cobyrinic acid a,c-diamide synthase	2.951778863
DP16_RS11400	chemoreceptor glutamine deamidase CheD	2.123279103
DP16_RS11455	betaine-aldehyde dehydrogenase	0.363677485
DP16_RS11705	beta-mannosidase	3.007720472
DP16_RS11710	N-acylglucosamine 2-epimerase	2.724971851
DP16_RS11715	fructokinase	2.835752241
DP16_RS11785	bifunctional phosphoribosyl-AMP cyclohydrolase/phosphoribosyl-ATP diphosphatase	0.246254946
DP16_RS11790	imidazole glycerol phosphate synthase cyclase subunit	0.127700975
DP16_RS11795	1-(5-phosphoribosyl)-5-[(5-phosphoribosylamino) methylideneamino]imidazole-4-carboxamide isomerase	0.147268194
DP16_RS11800	imidazole glycerol phosphate synthase subunit HisH	0.231537231
DP16_RS11805	bifunctional imidazole glycerol-phosphate dehydratase/histidinol phosphatase	0.280055192
DP16_RS11810	histidinol-phosphate transaminase	0.294877186
DP16_RS11815	histidinol dehydrogenase	0.443030227
DP16_RS11820	ATP phosphoribosyltransferase	0.436133828
DP16_RS13530	carbon storage regulator	2.009278707
DP16_RS14185	glutathione S-transferase	2.015208782
DP16_RS15035	alpha/beta hydrolase	2.019359987
DP16_RS15055	phosphatase	2.524646349
DP16_RS15060	Diaminobutyrate-2-oxoglutarate aminotransferase	2.077583577
DP16_RS15085	type I methionyl aminopeptidase	0.448587741
DP16_RS15455	DNA primase traC	2.667308905
DP16_RS15480	adenine methyltransferase	12.366258814
DP16_RS15515	catalase	0.412635332
DP16_RS16270	phosphoribosyl-dephospho-CoA transferase	3.169513160
DP16_RS16275	triphosphoribosyl-dephospho-CoA synthase MdcB	4.121058063
DP16_RS16280	malonate decarboxylase subunit epsilon	3.282040254
DP16_RS16285	Dicarboxylate carrier MatC domain protein	3.357663301
DP16_RS17100	integrase	2.177882406
DP16_RS17135	glycoside hydrolase	7.918573311
DP16_RS17465	integrase core domain protein	2.645454049
DP16_RS18245	diguanylate phosphodiesterase	2444.253612136

DP16_RS19860	resolvase	4.692493189
DP16_RS19890	AAA domain protein	6.663328858
DP16_RS19905	isoleucyl-tRNA synthetase	5.893245907
DP16_RS19930	DNA methylase N-4	3.422511827
DP16_RS19935	DNA methylase N-4	3.516898716
DP16_RS19970	terminase	4.805844658
DP16_RS19995	peptidase S49 family protein	4.070984505
DP16_RS20095	glycoside hydrolase	3.717412941
DP16_RS20100	ATP-dependent Lon protease	3.387595774
DP16_RS20105	alkaline phosphatase	2.633160194
DP16_RS20130	ATPase	2.365439361
DP16_RS20165	ATP-binding protein	2.734425895
DP16_RS21310	BioH protein	2.017890934
DP16_RS21425	methylisocitrate lyase	2.636025134
DP16_RS21980	L2 family class A beta-lactamase	0.405409761
DP16_RS23120	nucleotidyltransferase[Vibrioparahaemolyticu]	2.410683187
**Oxidation-Reduction process (24)**		
DP16_RS03125	oxidoreductase	0.398825283
DP16_RS04005	cioB	0.490516895
DP16_RS05560	dehydrogenase	2.005112423
DP16_RS05685	methylmalonate-semialdehyde dehydrogenase (acylating)	2.043399616
DP16_RS05705	3-hydroxyisobutyrate dehydrogenase	2.860785578
DP16_RS05710	Organic hydroperoxide resistance protein	2.199058904
DP16_RS06755	D-lactate dehydrogenase	0.296897506
DP16_RS06760	alpha-hydroxy-acid oxidizing enzyme	0.346768989
DP16_RS09095	ribonucleotide-diphosphate reductase	0.265130803
DP16_RS09100	ribonucleotide-diphosphate reductase subunit beta	0.420533949
DP16_RS09105	flavodoxin	0.388560409
DP16_RS09400	nitrate reductase subunit alpha	0.321956215
DP16_RS09405	nitrate reductase subunit beta	0.380763983
DP16_RS09410	nitrate reductase molybdenum cofactor assembly chaperone	0.346182978
DP16_RS09415	nitrate reductase A subunit gamma	0.356190415
DP16_RS09430	coproporphyrinogen III oxidase	0.445002776
DP16_RS09455	sulfite reductase subunit alpha	0.204030608
DP16_RS10195	oxidoreductase	2.986295967
DP16_RS10360	1,4-beta-D-glucan glucohydrolase	3.494442378
DP16_RS12965	putative short-chain dehydrogenase/reductase	0.462773765
DP16_RS13310	quercetin 2,3-dioxygenase	0.485912453
DP16_RS16240	malate:quinone oxidoreductase	0.403359989
DP16_RS16260	biotin-independent malonate decarboxylase subunit beta	2.198109909
DP16_RS18460	peroxiredoxin	0.290012243
**signal transduction (20)**		
DP16_RS01505	rod shape-determining protein RodA	2.402922329
DP16_RS06995	chemotaxis protein	2.638716749

DP16_RS09460	PAS sensor domain-containing protein	0.193198626
DP16_RS11055	chemotaxis protein	2.232129462
DP16_RS11065	histidine kinase	2.450562637
DP16_RS11100	chemotaxis protein CheW	2.537069212
DP16_RS11205	two-component system response regulator	2.279029760
DP16_RS11320	chemotaxis regulatory protein CheY	2.912407603
DP16_RS11325	chemotaxis protein	2.557752053
DP16_RS11330	chemotaxis protein CheA	2.840357818
DP16_RS11350	chemotaxis protein CheW	2.299734061
DP16_RS11355	STAS domain protein	2.471677223
DP16_RS11360	Fis family transcriptional regulator	2.170466428
DP16_RS11370	chemotaxis protein	2.457488269
DP16_RS11390	chemotaxis protein	2.445530755
DP16_RS19215	cold-shock protein	2.005290529
DP16_RS19775	serine/threonine kinase	0.490781281
DP16_RS21040	histidine kinase	2.348615962
DP16_RS22540	chemotaxis protein	2.072610253
DP16_RS22960	chemotaxis protein	2.888572399
DP16_RS20150	WYL domain-containing protein	2.006811219
**transcription regulation (15)**		
DP16_RS05675	RpoH suppressor	2.095497814
DP16_RS06950	heavy metal-responsive transcriptional regulator	0.477310873
DP16_RS09320	AraC family transcriptional regulator	2.352540132
DP16_RS09435	transcriptional regulator	0.374666962
DP16_RS10595	transcriptional regulator	0.469981898
DP16_RS11195 (FsnR)	DNA-binding response regulator	1.896370759
DP16_RS11200	RNA polymerase sigma-54 factor	3.085734337
DP16_RS11315	RNA polymerase sigma factor FliA	2.589700654
DP16_RS11450	transcriptional regulator BetI	0.404102825
DP16_RS12140	transcriptional regulator	2.777110984
DP16_RS17125	helix-turn-helix, Psq domain protein	7.408033716
DP16_RS19840	transcriptional regulator	3.768529572
DP16_RS19850	OST-HTH/LOTUS domain protein	3.251058124
DP16_RS20175	transcriptional regulator	3.670362282
DP16_RS22055	RNA polymerase subunit sigma	1.89637076
**Hypothetical protein (110)**		
DP16_RS02515	hypothetical protein	4.219766041
DP16_RS02530	hypothetical protein	3.014118601
DP16_RS02805	hypothetical protein	2.923498041
DP16_RS02825	hypothetical protein	2.795315917
DP16_RS02870	hypothetical protein	2.507397213
DP16_RS03250	hypothetical protein	2.399579675
DP16_RS03355	unnamed protein product	2.243843847
DP16_RS04680	hypothetical protein WJ66_02118	2.021444810

DP16_RS05555	hypothetical protein	3.268250169
DP16_RS05730	hypothetical protein	4.454197488
DP16_RS05735	hypothetical protein	2.344314467
DP16_RS05990	hypothetical protein	0.291679347
DP16_RS05995	hypothetical protein	0.411057879
DP16_RS06230	hypothetical protein	3.683922734
DP16_RS06620	DUF1338 domain-containing protein	2.126196077
DP16_RS06665	hypothetical protein	2.738159298
DP16_RS10590	hypothetical protein	0.478240151
DP16_RS10655	hypothetical protein	0.141073791
DP16_RS11190	hypothetical protein	2.893410583
DP16_RS11385	hypothetical protein	2.168537731
DP16_RS11415	hypothetical protein	2.290065868
DP16_RS13495	hypothetical protein	2.666304647
DP16_RS15430	hypothetical protein	2.780681913
DP16_RS15450	hypothetical protein	2.095910285
DP16_RS15470	hypothetical protein	5.313779459
DP16_RS15475	hypothetical protein	12.503010491
DP16_RS15485	hypothetical protein	14.065886803
DP16_RS15760	hypothetical protein	9999.396513345
DP16_RS16500	hypothetical protein	2.625632203
DP16_RS17090	hypothetical protein	4.264419650
DP16_RS17095	hypothetical protein	3.812669517
DP16_RS17110	hypothetical protein	10.971391706
DP16_RS17115	hypothetical protein	5.595097195
DP16_RS17130	hypothetical protein	10.002408393
DP16_RS17140	hypothetical protein	9.168874360
DP16_RS17145	hypothetical protein	6.826643728
DP16_RS17175	hypothetical protein	5.697394553
DP16_RS17415	hypothetical protein	3.245973878
DP16_RS17475	hypothetical protein	8697.699925809
DP16_RS17480	hypothetical protein	18.285652844
DP16_RS17490	hypothetical protein	2.220929495
DP16_RS17535	hypothetical protein	3.802999024
DP16_RS17540	hypothetical protein	3.779319080
DP16_RS17545	hypothetical protein	3.312785362
DP16_RS17550	hypothetical protein	2.545255707
DP16_RS17555	hypothetical protein	3.600867022
DP16_RS17560	hypothetical protein	6.043121738
DP16_RS17570	hypothetical protein	2.117966863
DP16_RS17575	hypothetical protein	5.110605538
DP16_RS17615	hypothetical protein	2.487578129
DP16_RS17725	hypothetical protein	2.082623550
DP16_RS17885	hypothetical protein	0.403924017

DP16_RS19080	hypothetical protein	7.271649194
DP16_RS19300	hypothetical protein	2.018011237
DP16_RS19830	hypothetical protein	4.955372047
DP16_RS19835	hypothetical protein	4.542568420
DP16_RS19845	hypothetical protein	4.092966642
DP16_RS19855	hypothetical protein	4.087068996
DP16_RS19865	hypothetical protein	4.002488115
DP16_RS19870	hypothetical protein	3.865171473
DP16_RS19875	hypothetical protein	5.224472241
DP16_RS19880	hypothetical protein	5.811719121
DP16_RS19885	hypothetical protein	7.315517020
DP16_RS19895	hypothetical protein	6.497283603
DP16_RS19900	hypothetical protein	7.527013977
DP16_RS19910	hypothetical protein	7.022524226
DP16_RS19915	hypothetical protein	8.168343091
DP16_RS19920	hypothetical protein	6.095217615
DP16_RS19925	hypothetical protein	9.299114053
DP16_RS19940	hypothetical protein	2.719404782
DP16_RS19960	hypothetical protein	2.431140929
DP16_RS19965	hypothetical protein	4.965684280
DP16_RS19975	hypothetical protein	3.362766213
DP16_RS19980	hypothetical protein	3.481054000
DP16_RS19985	hypothetical protein	3.000722518
DP16_RS20010	hypothetical protein	5.740018453
DP16_RS20015	hypothetical protein	4.568064190
DP16_RS20020	hypothetical protein	3.681952722
DP16_RS20025	hypothetical protein	2.849476423
DP16_RS20030	hypothetical protein [Cupriavidus sp. WS]	4.269410347
DP16_RS20035	hypothetical protein	4.646641212
DP16_RS20045	hypothetical protein	2.885775486
DP16_RS20050	hypothetical protein	3.325154083
DP16_RS20060	Uncharacterized conserved protein	3.073656746
DP16_RS20065	hypothetical protein DP16_399	4.571413211
DP16_RS20075	hypothetical protein	4.665185790
DP16_RS20080	hypothetical protein	3.697318817
DP16_RS20110	hypothetical protein	2.693235690
DP16_RS20115	hypothetical protein	3.212730087
DP16_RS20120	hypothetical protein	3.392490015
DP16_RS20135	hypothetical protein [Proteobacteria]	2.271809896
DP16_RS20140	hypothetical protein	2.245504519
DP16_RS20155	hypothetical protein	2.879037700
DP16_RS20160	hypothetical protein	2.191725952
DP16_RS20170	hypothetical protein	3.107365843
DP16_RS20180	hypothetical protein	4.447093504

DP16_RS20185	hypothetical protein	3.454779215
DP16_RS21305	conserved hypothetical protein	2.425855840
DP16_RS22040	hypothetical protein DP16_373	9.064682606
DP16_RS22045	hypothetical protein	2.711111969
DP16_RS22050	hypothetical protein	3.553844830
DP16_RS22060	hypothetical protein	3.733210208
DP16_RS22165	hypothetical protein	2.664331998
DP16_RS22345	hypothetical protein	4.376053672
DP16_RS22350	hypothetical protein	2.219284362
DP16_RS22365	hypothetical protein	2.416447220
DP16_RS23000	hypothetical protein	2.739917533
DP16_RS23215	hypothetical protein	8.439532082
DP16_RS23245	hypothetical protein	4.013466368
DP16_RS23295	hypothetical protein	3.264674962
